# Limiting Hearing Loss in Transgenic Mouse Models

**DOI:** 10.1523/ENEURO.0465-24.2025

**Published:** 2025-02-18

**Authors:** Travis A. Babola, Naomi Donovan, Sean S. Darcy, Catalina D. Spjut, Patrick O. Kanold

**Affiliations:** ^1^Department of Biomedical Engineering, Johns Hopkins University, Baltimore, Maryland 21205; ^2^Kavli Neuroscience Discovery Institute, Johns Hopkins University, Baltimore, Maryland 21205

**Keywords:** behavior, deaf, hearing loss, mouse, transgenic

## Abstract

Transgenic mice provide unprecedented access to manipulate and visualize neural circuits; however, those on a C57BL/6 background develop progressive hearing loss, significantly confounding systems-level and behavioral analysis. While outbreeding can limit hearing loss, it introduces strain variability and complicates the generation of complex genotypes. Here, we propose an approach to preserve hearing by crossing transgenic mice with congenic B6.CAST-*Cdh23^Ahl^*^+^ mice, which maintain low-threshold hearing into adulthood. Widefield and two-photon imaging of the auditory cortex revealed that 2.5-month-old C57BL/6 mice exhibit elevated thresholds to high-frequency tones and widespread cortical reorganization, with most neurons responding best to lower frequencies. In contrast, *Ahl+* C57BL/6 mice exhibited robust neural responses across tested frequencies and sound levels (4–64 kHz, 30–90 dB SPL) and retained low thresholds into adulthood. Our approach offers a cost-effective solution for generating complex genotypes and facilitates more interpretable systems neuroscience research by eliminating confounding effects from hearing loss.

## Significance Statement

Common C57BL/6 mice exhibit severe progressive hearing loss, which is a serious confound for systems-level and behavioral studies. While outbreeding to an unaffected strain can help, this is cost-prohibitive if multiple transgenes are introduced. We propose and validate a simple approach that preserves hearing in transgenic mice by crossing them with congenic B6.CAST-Cdh23*Ahl+* mice. Using widefield and two-photon imaging of the auditory cortex, we demonstrate that *Ahl+* C57BL/6 mice maintain robust neural responses across a range of frequencies and sound levels into adulthood. We also offer a rapid method to genotype offspring without the need to sequence. Our approach offers a cost-effective solution for generating complex genotypes while preserving hearing, thereby facilitating more accurate, reproducible, and interpretable neuroscience research.

## Introduction

Transgenic animal models have revolutionized our understanding of the brain and provided unprecedented access to cell-type–specific monitoring and manipulation. However, many commonly used tools for cell-type–specific manipulation, such as conditional knock-out alleles (International Mouse Knockout Consortium, 2007), fluorescent reporters (Madisen et al., 2010), and calcium indicators (Dana et al., 2014), are maintained in C57BL/6 mice, which exhibit progressive hearing loss (Mikaelian et al., 1974; Keithley et al., 2004; Zhang et al., 2013). This hearing deficit has been linked to a single-nucleotide polymorphism (SNP) in *Cdh23* at the *ahl* (age-related hearing loss) locus (Johnson et al., 1997). This gene encodes Cadherin23, a crucial component of the tip-link structure of the stereocilia in inner hair cells of the cochlea responsible for converting sound waves into neural activity (Noben-Trauth et al., 2003). Therefore, when using adult transgenic mice, researchers must remain aware that these mice progressively lose their ability to hear starting as early as postnatal day (P)21 (Zhang et al., 2013), acting as a potential confound in many experimental contexts. Progressive hearing loss is clearly problematic for auditory physiology but also poses challenges for experiments that rely on auditory cues to trigger behavior, such as reward-based learning tasks or maternal pup retrieval, where auditory perception is essential for normal behavioral outcomes.

Over the past decade, several strategies have been developed to mitigate age-related hearing loss in these mouse models. Commonly, transgenic animals are outbred to strains that carry *Cdh23*^Ahl+^ variants, producing F1 litters that are *Ahl+* (autosomal dominant) and carry the resulting transgene (Frisina et al., 2011; Romero et al., 2020; Liu and Kanold, 2021). One disadvantage of this strategy is that behavior and plasticity can be highly dependent on strain (Ranson et al., 2013; Kim et al., 2017; Sinclair et al., 2017; Sultana et al., 2019), making comparisons across studies difficult. Moreover, this outbreeding strategy works efficiently only when a single transgene is required. Generating complex transgenic animals, such as using a Cre and Cre-dependent reporter to study a particular cell population, becomes increasingly difficult, as all transgenes must be maintained on a single breeder. Researchers must also avoid Cre lines that result in germline recombination such as *CamKIIα-Cre*, *Emx1-Cre*, *PV-Cre*, *GFAP-Cre*, *Foxg1-Cre*, or *Six3-Cre* (Luo et al., 2020), which are commonly used to study neuronal and glial populations. When conditional knock-outs are required, this strategy becomes even more challenging, as knock-out alleles (or the Cre) must be outbred and made congenic by multiple rounds of backcrossing to achieve a pure genetic background. Achieving the gold standard of 10 or more generations of backcrossing takes 2–3 years if offspring are randomly selected, with modern speed congenic services taking five generations or 1.5 years (Wong, 2002). More recently, the SNP in C57BL/6 mice was corrected in a single generation using CRISPR/Cas9-mediated genome editing (Mianné et al., 2016), but this process requires considerable resources to design, generate, and screen animals and carries the additional risk of off-target gene editing. These approaches are impractical for most laboratories and a drain of resources for those that can afford it.

In this study, we demonstrate that crossing with a commercially available congenic strain, B6.CAST-Cdh23^Ahl+^/Kjn, which has been outbred and backcrossed to C57BL/6 for over 10 generations, allows for the rapid generation of transgenic animals expressing pan-neuronal GCaMP6s that carry *Ahl+*, therefore limiting the effects of progressive hearing loss. These animals can be genotyped using traditional PCR followed by a restriction enzyme digest, foregoing costly Sanger sequencing to detect *ahl* variants. Using widefield and two-photon imaging, we demonstrate that C57BL/6 mice carrying the *Ahl+* allele exhibit neural responses at thresholds similar to those observed in mice outbred to CBA/CaJ and retain low thresholds at 6 months of age, within the timeframe of most adult behavior and imaging studies. By identifying the advantages of this approach, we hope to enable better, more interpretable, and cost-effective experiments in neuroscience systems research.

## Materials and Methods

### Animals

Both male and female mice aged between 2 and 6 months were used for experiments. For the initial widefield imaging session, *ahl* B6 mice were (mean ± SD) 71 ± 17 d old, *Ahl+ B6* mice were 72 ± 18 d old, and *Ahl+* B6.CBA mice were 88 ± 9 d old. For 6-month-old widefield imaging session, *ahl* B6 mice were (mean ± SD) 191 ± 13 d old, *Ahl+* B6 mice were 196 ± 13 d old, and *Ahl+* B6.CBA mice were 188 ± 6 d old. For 2P imaging sessions, *ahl* B6 mice were (mean ± SD) 83 ± 17 d old, *Ahl+* B6 mice were 85 ± 24 d old, and *Ahl+* B6.CBA mice were 109 ± 10 d old.

We include sex as a factor for most analyses, which are reported if relevant. All animals were healthy (not-immunodeficient) and were only used for experiments detailed in this study. *Thy1-GCaMP6s* (GP4.3; Jax#, 024275) were crossed to *Cdh23*^Ahl/ahl^ heterozygotes [F1 offspring of B6.CAST-*Cdh23^Ahl+^*/Kjn (Jax#, 002756) and C57BL/6J (Jax#, 000664)] to generate *Ahl+* and control littermates. For *Ahl+* B6.CBA mice, F1 offspring of *Thy1-GCaMP6s* and *CBA/CaJ* mice were used.

Mice were group housed on a 12 h light/dark cycle and were provided food *ad libitum*. This study was performed in accordance with the recommendations provided in the Guide for the Care and Use of Laboratory Animals of the National Institutes of Health. All experiments and procedures were approved by the Johns Hopkins Institutional Care and Use Committee. All surgery was performed under isoflurane anesthesia, and every effort was made to minimize suffering.

### Genotyping *Cdh23* for *Ahl+* and *ahl* allelic variants

A KAPA2G HotStart kit (KK7352, Roche) was used for all genotyping. Briefly, DNA was extracted from tail clippings and amplified with PCR with the following primers: GTGCTGTTGGGCCTCCTTGC and GGGGTGGACCATGATCTATTTTGT. A 10 μl reaction volume was used, comprised of 5 μl 2X KAPA2G mix, 2 μl of primer mix (10 μM final volume of each primer), 2 μl of DNAase, RNAase-free H_2_O, and 1 μl of extracted DNA. HotStart was initiated with 2 min at 95°C, followed by 32 repeats of 95–63–72°C steps (denaturing–annealing–extension; 10 s each). A 2 min final extension at 72°C was followed by a 4°C hold. Finally, 0.5 μl (500 units) of BsrI was added to the reaction, incubated at 65°C for 15 min, and run in 1.8% agarose gel for ∼15 min at 150 V. Expected sizes are 284 and 664 base pairs for *ahl* and 948 bp for *Ahl+*.

### Cranial window installation

#### Animal preparation

Before surgery, mice were injected with dexamethasone (2 μg/g; delivered intramuscularly to the quadriceps of the hindpaw) to limit brain swelling. Animals were anesthetized with isoflurane (Fluriso, VetOne) using a calibrated vaporizer. Anesthesia was induced at 4% isoflurane for 5 min and maintained at 1.5–2% for the duration of the surgery (∼1 h). Body temperature was maintained at 36.5°C using an internal thermometer and feedback-controlled heating blanket (Harvard Apparatus 50-7212). Hair was removed from the scalp using a 5 min application of Nair hair removal cream and thorough cleaning of the surgical area with three successive Betadine and ethanol rinses. All surgical tools were sterilized before the first incision.

#### Cranial window and headpost implantation

Surgical scissors were used to remove the scalp from the dorsal aspect of the skull, starting slightly anterior to the ears to the bregma suture, with lateral incisions ∼3 mm to the right of and 5 mm to the left of the sagittal suture. The left temporalis muscle was retracted laterally to expose the squamosal suture and the posterior aspect of the jugal bone. Surgical calipers were used to lightly etch a 3.5 mm square encompassing the auditory cortex, with the posterior edge defined by the lambdoid suture and the lateral edge defined by the squamosal suture. The “fold” of the parietal bone, running in the anterior to posterior direction, was located roughly in the middle of this square. A 1 mm dental drill bit was used to bore a circular shape, followed by more delicate removal using a 0.5 mm bit. The skull was carefully removed, and a modified coverslip consisting of a 4 mm circular cover glass adhered to a 3 mm circular coverslip with optical glue was adhered to the skull initially with cyanoacrylate glue and permanently secured with dental cement, along with the headpost.

#### Postoperative care

Mice were kept on the surgical blanket until major muscle movements were observed and then transferred to a recovery cage placed on a circulating water blanket maintained at 36°C. Carprofen (Rimadyl; 5 mg/kg, i.p.) was administered immediately after the surgery and continued for the next 3 d. DietGel (Clear H20) was provided in the cage during this recovery period.

### Sound presentation and calibration

Auditory stimuli consisted of sinusoidal amplitude-modulated tones (500 ms duration, 10 Hz modulation frequency) with a cosine gate applied to the first 10 ms to minimize sprectral artifacts. Tones were presented at frequencies ranging from 4 to 64 kHz and sound pressure levels of 90, 70, 50, and 30 dB SPL, designed to span the hearing range of mice. This limited set of stimuli was chosen to reduce head-fixation duration and associated stress, which could influence neural state and activity. Stimuli were generated and delivered using a custom MATLAB script interfacing with an RX6 Multifunction Processor (Tucker-Davis Technologies) and played through an electrostatic speaker and driver (ES1 and ED1, Tucker-Davis Technologies). Each tone was calibrated to a 90 dB SPL reference using a Brüel and Kjær ultrasonic microphone.

### Widefield imaging

Mice were head-fixed in a holder under a custom widefield microscope. The field of view was illuminated with a 470 nm LED (M470L3, Thorlabs) through a 4× air objective (UplanSApo 4×/0.16, Olympus) mounted at ∼45° from vertical. The light path was separated via a low-pass dichroic mirror (MD499-FITC, Thorlabs) to illuminate the sample, and the light was collected via a sCMOS camera (pco.edge 4.2, Excelitas Technologies) following emission filter (AT535/40m, Chroma) and tube lens focusing (AC508-150A, Thorlabs). The camera and sound stimuli were triggered externally (USB-6259, National Instruments) at 30 Hz. Images were digitized at 330 × 330 pixels that encompassed the entire cranial window (∼3 mm^2^).

### Widefield data processing and analysis

Suite2p was used to register (nonrigid, 128 × 128 block size) widefield images. Default parameters were used except for the sampling rate (fs), which was changed to 30 to match our acquisition parameters. Images were then downsampled from 330 pixels^2^ to 100 pixels^2^ to hasten further calculations. Raw fluorescence traces were then unmixed, forming a matrix of size *X *× *Y* × *T *× *F *× *A *× *R*, where *X* is the width of the frame in pixels (100), *Y* is the height of frame in pixels (100), *T* is the time of the trial period (75 frames), *F* is the number of frequencies (5), *A* is the number of attenuations or sound levels (4), and *R* is the number of repeats (10).

**Figure 1.**
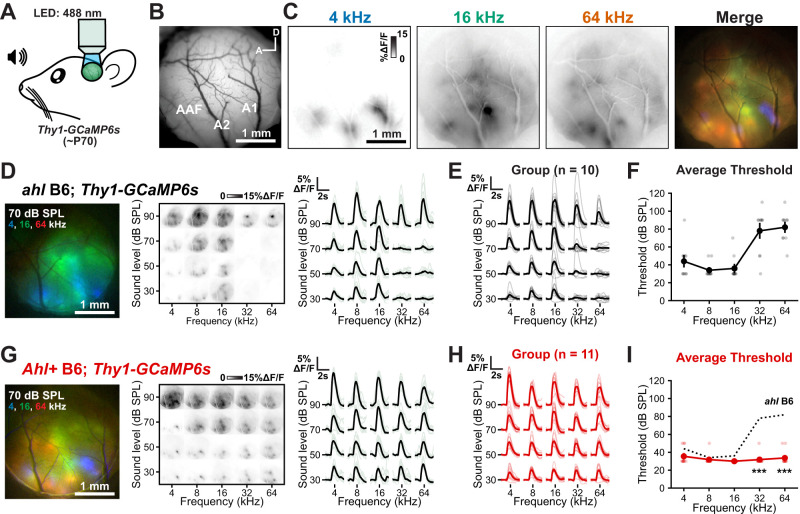
Widefield imaging of sound-evoked responses in the auditory cortex. ***A***, Schematic of widefield imaging in the auditory cortex of adult (∼P70) *Thy1-GCaMP6s* mice. ***B***, Representative image of the cranial window and the field of view captured for analysis. ***C***, Fluorescence responses to pure tones indicated at 70 dB SPL. Right, Merged fluorescence responses to pure tones highlight cortical topography. Blue, green, and red channels indicate responses to 4, 16, and 64 kHz, respectively. ***D***, Left, Merged fluorescence responses to 4 (blue), 16 (green), and 64 kHz (red) at 70 dB SPL in *ahl* B6; *Thy1-GCaMP6s* mice. Middle, The plot of fluorescence changes over the imaging field to varying frequency (*x*-axis) and sound levels (*y*-axis). Right, The plot of fluorescence during sound presentation from an individual mouse; gray lines are individual trials; the black line is the average. ***E***, The plot of fluorescence during sound presentation from all *ahl* B6; *Thy1-GCaMP6s* mice; gray are individual mice; black is the group average; *n* = 10 mice. ***F***, The plot of average fluorescence detection threshold for each frequency presented. Light individual markers represent individual mice. If there was no observable response at 90 dB SPL, the threshold was defined as 110 dB SPL. ***G–I***, Similar to ***D***–***F***, but for *Ahl+* B6; *Thy1-GCaMP6s* mice. Three-way ANOVA (threshold, sex, and genotype reported in text), followed by post hoc *t* tests with Benjamini–Hochberg FDR correction. ***p < 0.001. Extended Data Figure 1-1 extends this analysis for *Ahl+* B6.CBA; *Thy1-GCaMP6s* mice.

The baseline (*F*_o_) was calculated by averaging the 30 frames (1 s) prior to tone onset for each trial. This baseline was used to normalize each pixel's signal by calculating Δ*F*/*F* as (*F* − *F*_o_) / *F*_o_. For each tone and sound level response, we averaged the window between 10 and 15 frames after tone onset and used this as the sound response amplitude (visualized in images Fig. 1*D*,*G*). We then averaged only pixels with Δ*F*/*F* in the 99th percentile (visualized as traces in Fig. 1*D,G*). The threshold was calculated based on these responses, using a paired *t* test between the baseline and response amplitude, with *α* = 0.05 and no correction for multiple comparisons. Thresholds were defined as the sound with the first statistically significant response or 110 dB SPL if there was no detectable response at the highest sound level presented.

10.1523/ENEURO.0465-24.2025.f1-1 Figure 1-1 Widefield imaging in *Ahl* *+* B6.CBA mice reveals low threshold responses to a broad range of frequencies. **A,** (left) Merged fluorescence responses to 4 (blue), 16 (green), and 64 kHz (red) at 70  dB SPL in P70 *Ahl* *+* B6.CBA; *Thy1-GCaMP6  s* mice. (middle) Plot of fluorescence changes over the imaging field to varying frequency (x-axis) and sound levels (y-axis). (right) Plot of fluorescence during sound presentation from an individual mouse. Grey traces are individual trials, black traces are the average. **B,** Plot of fluorescence during sound presentation from all *Ahl* *+* B6.CBA; *Thy1-GCaMP6  s* mice. Light traces are individual mice, dark traces are the group average, n = 7 mice. **C,** Plot of average fluorescence detection threshold for each frequency presented, n = 7 mice. Three-way ANOVA (frequency: *F(4,110)* = 16.2*, p* = 1.8e-10; sex: *F(1,110)* = 4.76*, p* = 3.1e-02; genotype: *F(2,110)* = 61.7*, p* = 1.1e-18; interaction: *F(22,110)* = 5.13*, p* = 3.2e-09) followed by planned comparisons with t-tests controlled with Benjamini-Hochberg FDR. **: *p* < 0.01, ***: *p* < 0.001. **D,** (left) Plot of fluorescence changes in 2.5-month-old *Thy1-GCaMP6  s* (*Ahl* *+* B6.CBA) mice over the imaging field to varying frequency (x-axis) and sound levels (y-axis). (right) Plot of fluorescence during sound presentation across animals; grey traces are individual mice, black traces are the average, n = 5 mice. **E,** (left) Plot of fluorescence changes in 6-month-old *Thy1-GCaMP6  s* (*Ahl* *+* B6.CBA) mice over the imaging field to varying frequency (x-axis) and sound levels (y-axis). (right) Plot of fluorescence during sound presentation across animals; grey traces are individual mice, black traces are the average, n = 5 mice. **F,** Plot of average threshold as a function of frequency and time point. Dashed lines indicate measurements at 2.5 months, solid lines indicate measurements at 6 months, n = 5 mice. Three-way ANOVA (frequency: *F(4,30)* = 4.80*, p* = 4.1e-3; timepoint: *F(1,30)* = 10.8*, p* = 2.6e-3; sex: *F(1,30)* = 16.2*, p* = 3.6e-4, interaction: *F(13,30)* = 7.15*, p* = 5.0e-6) with post-hoc paired t-tests with Benjamini-Hochberg FDR correction. n.s.: not significant. Download Figure 1-1, TIF file.

### Two-photon imaging

Mice were head-fixed in a holder under an Ultima 2Pplus microscope (Bruker). This microscope features a rotatable objective to achieve imaging of the auditory cortex with animals in a normal stationary position. A 920 nm excitation laser (Chameleon Discovery NX, Coherent) was used in conjunction with a 16× objective (CFI75 LWD 16X W, Nikon) and resonant-galvo scanning to capture images at ∼15 Hz at 1,024 pixel^2^ resolution, resulting in a field of view of ∼1.1 mm^2^ that was targeted to the primary auditory cortex with widefield imaging maps (Fig. 1*C*). The frame out signal from the microscope was used to trigger sound stimuli.

### Two-photon data processing and analysis

#### Registration, fluorescence extraction, and DF/F calculation

Suite2p (Pachitariu et al., 2017) was used to register (nonrigid, 128 × 128 block size) and extract fluorescence from regions of interest (ROIs) and surrounding neuropil. Default parameters were used except for the sampling rate (fs), which was changed to 15 to match our acquisition parameters.

For each identified ROI, the raw fluorescence signal over time, $F_{\mathrm{ROI}}$, was extracted. The fluorescence signal used for analysis, F, was calculated by performing neuropil subtraction on the raw fluorescence signals of each ROI, $F=F_{\mathrm{ROI}}-(\alpha \times F_{\mathrm{NP}})$, where α=0.7, to decrease the contamination from neuropil fluorescence. To correct for slow drifts that occur over the length of the imaging session, we calculated the baseline (*F*_o_) using a moving-window approach. The baseline was defined as the 10th percentile of fluorescence values within a window of 500 frames with 50 frame steps. The resulting baseline was linearly interpolated to match the size of the original input. Finally, this baseline was used to normalize the neuronal signal by calculating Δ*F*/*F* as (*F* − *F*_o_)/*F*_o_.

#### Determination of sound-responsive neurons

To determine if a neuron was sound-responsive, we created a linear model to explain the signal amplitude using a response variable that encoded the signal phase (baseline, onset, or offset), the frequency, the attenuation, and the interactions between these variables [signal_amp ∼ *C*(response_variable) + *C*(frequency) + *C*(attenuation) + *C*(frequency):*C*(attenuation):*C*(response_variable)]. The response amplitude was calculated as the mean response during the 1 s before the sound was played (baseline), between Frames 5 and 9 after the tone onset (onset, ∼330–600 ms after the tone onset) and between Frames 5 and 9 after the tone offset (offset). We considered the neuron sound-responsive if the response variable explained the variance at an *α* < 0.01, following criteria used in previous studies (Winkowski and Kanold, 2013; Bowen et al., 2020). If the response variable was significant at this level, we performed post hoc *t* tests with Benjamini–Hochberg correction to compare the means of each response phase. If the onset amplitude was significantly higher than the baseline amplitude, the neurons were classified as “Onset” neurons. If the offset amplitude was significantly higher than the onset amplitude, the neurons were classified as “Offset” neurons. If both properties were present, the neuron was classified as an “Onset/Offset” neuron. For calculating the proportion of sound offset-responsive neurons, both “Offset” and “Onset/Offset” neurons were grouped as “Offset” neurons.

#### Best frequency, characteristic frequency, and bandwidth calculation

For all calculations described in this section, only the onset response amplitudes for “Onset” and “Onset/Offset” neurons were used. For every frequency and attenuation level, we performed post hoc *t* tests with multiple comparisons controlled via Benjamini–Hochberg procedure with a false discovery rate (*α* = 0.2). Only frequency and sound levels that showed a statistically significant response were included for subsequent analysis.

Bandwidth was defined as log_2_(*f*_max_ / *f*_min_), with *f*_max_ and *f*_min_ representing the maximum and minimum frequency with a significant response. Neurons with noncontinuous frequency responses (i.e., multiple peaks) were excluded from the analysis. Best frequency was defined as the frequency with the highest amplitude response, regardless of the sound level. Characteristic frequency was defined as the frequency with the highest amplitude response at the lowest sound level.

#### Signal and noise correlations

Signal and noise correlations were calculated following methods from previous studies (Winkowski and Kanold, 2013; Bowen et al., 2020). To measure signal correlations, we first averaged Δ*F*/*F* responses across all repeats for each frequency and sound level. We calculated the correlation coefficient over a 2 s window (30 frames) after the tone onset for each frequency and sound level between all pairs of sound-responsive neurons. These correlation values were then averaged to obtain a signal correlation value for each neuron pair. Finally, we averaged the correlation values across all pairs to get the overall signal correlation for each animal.

To measure noise correlations, which assess the correlations between neurons that are independent of their response to the sound, we first averaged Δ*F*/*F* responses across all repeats for each frequency and sound level and then subtracted this from each repeat. For each neuron pair, we computed the correlation coefficient on a repeat-by-repeat basis over a 1 s window (30 frames) after the tone onset. Finally, we averaged these correlation coefficients across all neuron pairs, frequency levels, sound levels, and repeats to obtain the overall noise correlation for each animal.

#### Linear discriminant analysis

Δ*F*/*F* traces were unmixed, forming a matrix of size *N *× *T *× *F *× *A *× *R*, where *N* is the number of neurons, *T* is the time of the trial period (75 frames), *F* is the number of frequencies (5), *A* is the number of attenuations or sound levels (4), and *R* is the number of repeats (10). For each trial, we calculated the average response over a 1 s (15 frames) following tone onset, resulting in an *N *× 1 × *F *× *A *× *R* array that was then reshaped into an *N *× (*F *× *A *× *R*) array.

The (*F *× *A *× *R*) dataset was randomly split into 90% training and 10% test data. For each split, principal component analysis was fit on the training subset, and linear features explaining at least 90% of the data variance extracted with this fit were used to train a linear discriminant analysis (LDA) classifier to classify neural trials by the corresponding tone and sound levels. The PCA features and LDA classifier were deployed on the testing subset to evaluate classification performance. This procedure was repeated 10 times to cover the entire dataset, and the average accuracy was reported. Full implementation details are available in the provided code.

### Quantification and statistical analysis

All statistics and corrections for multiple comparisons were performed in Python with the statsmodels (for linear models) and scipy (for post hoc testing) packages. All statistical details, including the exact value of *n*, what *n* represents, and which statistical test was performed, can be found in the figure legends and/or within the figure panels. Data in plots are presented as mean ± standard error of the mean, unless indicated otherwise. For single comparisons, significance was defined as *p* ≤ 0.05. When multiple comparisons were made, the Benjamini–Hochberg correction was used to adjust *p* values accordingly to lower the probability of Type 1 errors. For multiple condition datasets, an *N*-way ANOVA analysis was performed and, if relevant variables were statistically significant, followed by *t* test comparison with Benjamini–Hochberg correction.

#### Data and code availability

Files generated following suite2p preprocessing (fluorescence, classification, neuropil fluorescence, etc.) and source code for analysis and figure generation are available at https://zenodo.org/records/13773210 (code) and https://zenodo.org/records/13761406 (data), respectively. Any additional information required to reanalyze the data reported in this paper is available from the lead contact upon request.

## Results

### Outbreeding transgenic mice limits early hearing loss

A majority of transgenic mice used in modern neuroscience studies are maintained on the C57BL/6 background, a strain known to exhibit signs of progressive hearing loss as early as P21 (Mikaelian et al., 1974; Zhang et al., 2013). One such line is *Thy1-GCaMP6s* (referred to here as *ahl* B6, as it carries two copies of the recessive *ahl* allele), which expresses the genetically encoded calcium indicator GCaMP6s across most excitatory neurons and is widely used to study circuits in vivo (Dana et al., 2014). To determine if these mice exhibit symptoms of early hearing loss, we installed cranial windows and performed widefield imaging of the auditory cortex in awake mice (∼2.5 months old; see Materials and Methods for details) while presenting pure tones encompassing their hearing range [4–64 kHz in octave steps, 30–90 dB SPL (decibels, sound pressure level) in 20 dB SPL steps; Fig. 1*A*,*B*]. Pure tones resulted in visible fluorescence increases within discrete spatial domains of the auditory cortex; low-frequency tones (4 kHz) presented at moderate sound levels (50–70 dB SPL) typically activated three major areas, consistent with A1, secondary auditory cortex (A2), and anterior auditory field (AAF) functional designations (Liu et al., 2019; Romero et al., 2020), while higher frequencies activated complex spatial patterns with frequencies advancing from posterior to anterior in A1, dorsal to ventral in A2, and anterior to posterior–ventral in AAF (Fig. 1*C*). In general, *ahl* B6 mice exhibited robust, low-threshold responses following presentation of 4, 8, and 16 kHz tones (mean ± SD, 40 ± 20 dB SPL, 30 ± 10 dB SPL, and 40 ± 10 dB SPL, respectively; Fig. 1*D*–*F*), consistent with auditory brainstem responses (ABRs; Ison and Allen, 2004; Johnson et al., 2017). However, responses to higher-frequency tones (32 and 64 kHz) at low sound levels were undetectable in most (9 out of 10) mice (Fig. 1*F*). Responses to 32 and 64 kHz tones were elevated (mean ± SD, 80 ± 30 dB SPL and 80 ± 20 dB SPL, respectively), indicating a loss of hearing sensitivity at these frequencies (Fig. 1*F*). These data indicate that even at a young age, high-frequency hearing is significantly impaired.

To limit hearing loss in transgenic mice, a common strategy is to outbreed them to a strain that carries the *Ahl+* allele, such as CBA/CaJ (Liu et al., 2019; Bowen et al., 2020). Indeed, we observed robust widefield calcium responses to all frequencies and attenuations tested in F1 offspring using this breeding strategy [(CBA/Ca × C57BL/6J)F1, referred to here as *Ahl+* B6.CBA; Extended Data Fig. 1-1]. While this approach is tractable in animals carrying a single allele, generating complex transgenic animals, such as conditional knock-outs, is impossible while maintaining a consistent genetic background without generating congenic animals.

To bypass these difficulties, we used a commercially available congenic strain, *B6.CAST-Cdh23^Ahl+^/Kjn*, which has already been outbred and backcrossed to C57BL/6J for over 10 generations while selecting for the *Ahl+* allele. Similar to *Ahl+* B6.CBA mice, breeding *Thy1-GCaMP6s* mice to *B6.CAST-Cdh23^Ahl+^/Kjn* mice produced offspring (*Ahl+* B6) that exhibited neural responses to tones across all frequencies and attenuations tested (Fig. 1*G*,*H*). The tuning curve appeared flat rather than the expected U shape, likely due to the limited number of sound levels (30–90 dB SPL, 20 dB steps) used in this design, which prioritized minimizing head-fixation duration for awake mice while spanning their broad hearing range. Complete audiograms for this strain and similar *Ahl+* B6 congenic strains have been previously reported (Keithley et al., 2004; Vázquez et al., 2004; Kane et al., 2012; Mock et al., 2016; Johnson et al., 2017; Burghard et al., 2019), indicating that these mice exhibit U-shaped audiograms.

To compare auditory thresholds across all frequencies, genotypes, and sexes, we performed a three-way ANOVA, which revealed significant effects for all main factors: frequency (*F*_(4,110)_ = 16.2; *p* < 0.001), sex (*F*_(1,110)_ = 4.75; *p* = 0.031), and genotype (*F*_(2,110)_ = 61.7; *p* < 0.001), as well as their interactions (*F*_(22,110)_ = 5.1; *p* < 0.001). Although sex was a weakly significant factor, post hoc testing revealed no statistically significant differences between sexes within each genotype (*t* tests with corrected *p* value, *Ahl+* B6, *p* = 0.097; *Ahl+* B6.CBA, *p* = 0.462; *ahl* B6; *p* = 0.53). In contrast, frequency and genotype were very strong predictors. The *ahl* B6 mice exhibited higher thresholds at 32 and 64 kHz compared with both *Ahl+* B6 (mean ± SD, 32 ± 5 and 34 ± 8 dB SPL for 32 and 64 kHz, *t* test with corrected *p* < 0.001 and *p* < 0.001, respectively) and *Ahl+* B6.CBA mice (30 ± 0 and 33 ± 8 dB SPL for 32 and 64 kHz, *t* tests with corrected *p* = 0.001 and *p* < 0.001, respectively; Fig. 1*I* and Extended Data Fig. 1-1*C*). These data indicate that the *Ahl+* locus is critical for preserving high-frequency hearing, consistent with previous findings (Johnson et al., 2017). By implementing a simple cross to B6.CAST*-Cdh23^Ahl+^*/Kjn, our strategy enables the rapid generation of complex transgenic mice with limited hearing loss.

### C.573A variant introduces a restriction enzyme site that allows for fast genotyping without sequencing

**Figure 2.**
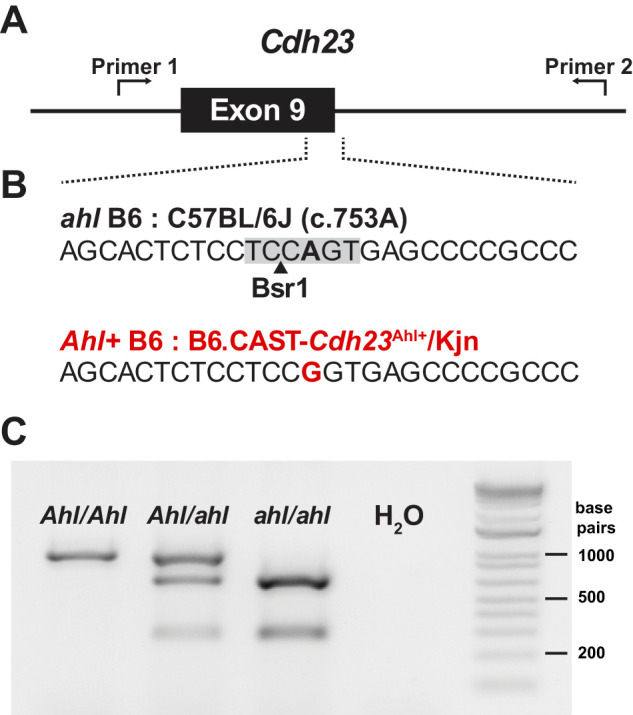
Genotyping *Cdh23* SNP responsible for hearing loss in C57BL/6 mice. ***A***, Schematic of *Cdh23* locus (Exon 9), where C57BL/6 mice exhibit a SNP. Primers are designed such that the SNP is offset from the center of the amplified region. ***B***, Sequence of the region denoted in ***A*** from C57BL/6 (top) and B6.CAST-*Cdh23*^Ahl+^/Kjn (bottom) mice. A BsrI restriction enzyme site is present in C57BL/6. ***C***, An image of gel following PCR amplification and BsrI restriction enzyme digest of DNA region shown in ***A*** in mice carrying zero, one, or two *ahl* alleles.

To maintain and generate new breeders and offspring carrying the *Ahl+* allele, we developed a rapid genotyping method. Typically, detecting SNPs like the c.573A variant (*ahl*) in C57BL/6 mice involves amplifying the region with PCR and subsequently submitting the sample for sequencing (Fig. 2*A*). However, the B6 *ahl* variant introduces a BsrI restriction enzyme site (Fig. 2*B*). By offsetting the PCR primers (Fig. 2*A*), we found that amplifying an ∼950 base pair region of *Cdh23* (Exon 9) and then digesting with BsrI can differentiate between mice with zero, one, or two copies of the recessive *ahl* allele. In *Ahl+* homozygous mice (*Cdh23*^Ahl/Ahl^), this method produces a single, uncut band of 948 base pairs (Fig. 2*C*). In *ahl* homozygous mice (*Cdh23*^ahl/ahl^), the DNA is cut by BsrI, resulting in bands at 284 and 664 base pairs. Heterozygous mice exhibit all three bands (Fig. 2*C*). This procedure takes ∼2 h, does not require DNA purification, and can be performed with a standard thermocycler (see Materials and Methods), offering a quick, efficient, and accessible alternative to sequencing or outsourcing to distinguish *Cdh23* variants.

### Two-photon imaging reveals diminished responses to high-frequency tones in *ahl* B6 mice

**Figure 3.**
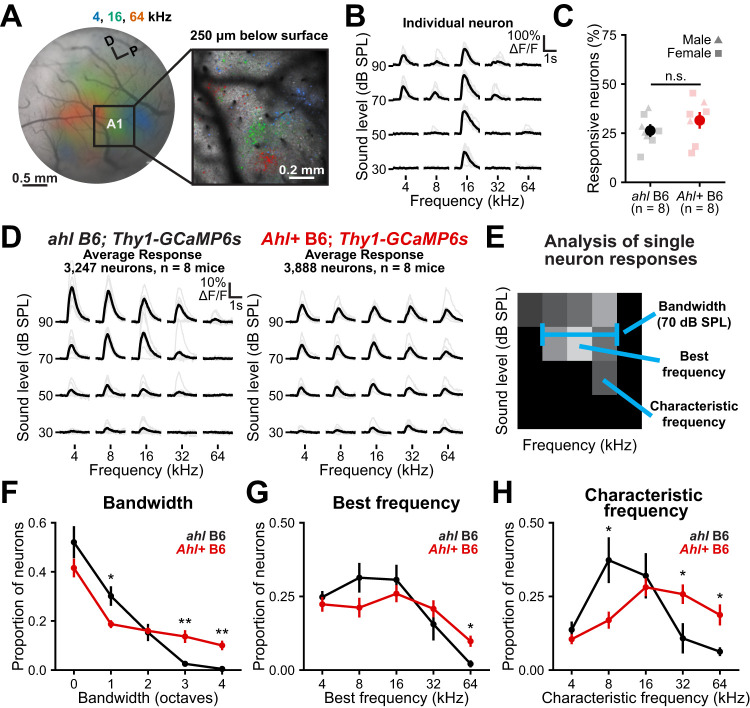
Two-photon imaging of sound-evoked responses in the auditory cortex. ***A***, Left, Schematic of widefield imaging in the auditory cortex of adult (P60) *Ahl+* B6; *Thy1-GCaMP6s* mice with overlaid responses to 70 dB SPL tones. Right, Two-photon imaging of the primary auditory cortex (A1, 250 µm from pial surface) with overlaid responses to 70 dB SPL tones. ***B***, Fluorescence traces from an individual neuron within the field of view depicted in ***A*** as a function of frequency (*x*-axis) and sound level (*y*-axis). Gray traces are individual trials; black traces are the trial averages. ***C***, The plot of the proportion of sound-responsive neurons within each genotype. Light markers indicate individuals and dark markers are mean ± SEM; *n* = 8 mice for each genotype. Two-way ANOVA, genotype, *F*_(2,15)_ = 2.42; *p* = 0.12; sex, *F*_(1,15)_ = 1.95; *p* = 0.18; interaction, *F*_(2,15)_ = 0.00; *p* = 0.99. n.s., not significant. ***D***, Average response across all sound-responsive neurons. Gray traces represent individual mice, and the black trace represents the group average. A total of 16 mice were analyzed (*n* = 8 per genotype). In ahl B6 mice, 3,247 total sound-responsive neurons were analyzed (mean ± SD, 406 ± 190 neurons per animal), while in *Ahl+* B6 mice, 3,888 total sound-responsive neurons were analyzed (mean ± SD, 486 ± 258 neurons per animal). Two-way ANOVAs revealed no significant effects of genotype or sex on either the total number of detected neurons (genotype, *F*_(1,12)_ = 0.0006; *p* = 0.980; sex, *F*_(1,12)_ = 0.083; *p* = 0.778; interaction, *F*_(1,12)_ = 1.32; *p* = 0.273) or the number of sound-responsive neurons (genotype, *F*_(1,12)_ = 0.665; *p* = 0.431; sex, *F*_(1,12)_ = 0.379; *p* = 0.550; interaction, *F*_(1,12)_ = 0.528; *p* = 0.481). ***E***, Schematic indicating measurements reported in ***F–H***. The color black indicates the response of an individual neuron to different frequencies and sound levels, with lighter colors indicating larger responses. The bandwidth is defined as the width in octaves separating the highest and lowest responding frequencies; a bandwidth of 0 indicates the neuron was responsive to a single tone. Best frequency is defined as the frequency with the highest amplitude response, regardless of the sound level. Characteristic frequency is defined as the frequency eliciting the highest amplitude response at the lowest sound level. ***F–H***, Plots of the proportion of neurons as a function of bandwidth at 70 dB, best frequency, and characteristic frequencies across genotypes. Markers are mean ± SEM; *n* = 8 mice for each genotype. Two-way ANOVAs with genotype and measurement, followed by post hoc *t* tests with Benjamini–Hochberg FDR correction. *p < 0.05; **p < 0.01; ***p < 0.001. Extended Data Figures 3-1 and 3-2 extend this analysis to include comparisons to *Ahl+* B6.CBA; *Thy1-GCaMP6s* mice.

To define the response properties of individual neurons, we performed two-photon imaging within the auditory cortex in awake mice. Using the tonotopic maps identified with widefield imaging as a reference (Fig. 3*A*), the imaging field of view encompassed the primary auditory cortex (A1; ∼1.1 mm^2^). We imaged 250 µm below the pial surface, anatomically at the bottom of Layer III and the top of Layer IV (de Vries et al., 2020). Neurons within A1 exhibited spatially organized responses to tones (Fig. 3*A*), with many that were broadly responsive to louder tones and selective to quieter tones (Fig. 3*B*), characteristic of “V-shaped” tuning. Overall, there were no significant differences in the proportion of sound-responsive neurons in *Ahl+* B6 or B6.CBA mice compared with *ahl* B6 mice (Fig. 3*C*; Extended Data Fig. 3-1*A*). However, the proportions of neurons responding to sound offset were significantly higher in *Ahl+* B6 and B6.CBA mice compared with *ahl* B6 mice (Extended Data Fig. 3-2*A*), similar to *Ahl+* B6.CBA mice in previous studies (Bowen et al., 2020). When examining the average tuning curve across all sound-responsive neurons, neuronal response properties were remarkably consistent with widefield imaging. The *ahl* B6 mice lacked responses to high-frequency tones (32 and 64 kHz) at lower sound levels, while *Ahl+* B6 mice exhibited responsiveness across all frequencies and sound levels (Fig. 3*D*). These data indicate that *ahl* B6 mice lack neurons responding to high frequencies across A1 and that widefield imaging serves as a reasonable proxy for assessing general cortical responsiveness to sounds.

10.1523/ENEURO.0465-24.2025.f3-1 Figure 3-1 Neuronal responses in *Ahl* *+* B6.CBA are similar to *Ahl* *+* B6 mice. **A,** Plot of the proportion of sound-responsive neurons within each genotype. Light markers indicate individuals, dark markers are mean ± SEM, n = 8 mice for *ahl* and *Ahl* *+* B6 mice, n = 5 mice for *Ahl* *+* B6.CBA mice. Two-way ANOVA (genotype: *F(2,15)* *=* 2.42, *p* = 0.12; sex: *F(1,15)* = 1.95*, p* = 0.18, interaction: *F(2,15)* = 0.00*, p* = 0.99). n.s.: not significant. **B,** Plots of the proportion of neurons as a function of bandwidth, best frequency, and characteristic frequencies across genotypes. Points are mean ± SEM, n = 8 mice for *ahl* and *Ahl* *+* B6 mice, n = 5 mice for *Ahl* *+* B6.CBA mice. Two-way ANOVAs with genotype and measured characteristic, followed by post-hoc t-tests with Benjamini-Hochberg FDR correction. *: *p* < 0.05, **: *p* < 0.01, ***: *p* < 0.001. Comparisons between *Ahl* *+* B6 and *ahl* B6 are indicated with red stars, *Ahl* *+* B6.CBA and *ahl* B6 with yellow stars, and *Ahl* *+* B6 and *Ahl* *+* B6.CBA with blue stars. **C,** Exemplar characteristic frequency maps and assigned areas for *Ahl* *+* B6.CBA mice. White areas on the map indicate regions where no characteristic frequency was assigned due to a lack of neurons within the area. **D,** Plot of normalized area as a function of characteristic frequency and genotype. Light lines are individual animals, dark lines are mean ± SEM, n = 8 mice for *ahl* and *Ahl* *+* B6, *n* = 5 for *Ahl* *+* B6.CBA. Two-way ANOVA (characteristic frequency: *F(1,36)* = 72.98*, p* < 0.001; genotype: *F(2,36)* *=* *0.08, p* = 0.92; interaction: *F(2,36)* = 34.6, *p* < 0.001), followed by post-hoc t-tests with Benjamini-Hochberg FDR correction. n.s: not significant, **: *p* < 0.01, ***: *p* < 0.001. **E,** (left) Low-dimensional representation (t-SNE) of neuronal population response from an individual *Ahl* *+* B6.CBA mouse with each marker representing a single trial. (right) Confusion matrix of classifier performance from a single animal. **F,** Plot of overall classifier performance as a function of frequency, n = 8 mice for *ahl* and *Ahl* *+* B6 mice, n = 5 mice for *Ahl* *+* B6.CBA mice. Three-way ANOVA (genotype: *F(2,360)* = 76.7*, p* < 0.001; frequency: *F(4,360)* = 15.2*, p* < 0.001; sound level: *F(3,360)* = 47.8*, p* < 0.001; interaction: *F(50,360)* = 2.89*, p* < 0.001), all attenuation levels included for each frequency in post-hoc t-tests with Benjamini-Hochberg FDR correction. *: *p* < 0.05, **: *p* < 0.01, ***: *p* < 0.001. Comparisons between *Ahl* *+* B6 and *ahl* B6 are indicated with red stars, *Ahl* *+* B6.CBA and *ahl* B6 with yellow stars, and *Ahl* *+* B6 and *Ahl* *+* B6.CBA with blue stars. **G,** Plot of classifier performance as a function of frequency (x-axis) and sound level (plots arranged from highest sound level to lowest sound level). n = 8 mice for *ahl* and *Ahl* *+* B6 mice, n = 5 mice for *Ahl* *+* B6.CBA mice. The same three-way ANOVA is reported in **F,** post-hoc t-tests with Benjamini-Hochberg FDR correction. *: *p* < 0.05, **: *p* < 0.01, ***: *p* < 0.001. Comparisons between *Ahl* *+* B6 and *ahl* B6 are indicated with red stars, *Ahl* *+* B6.CBA and *ahl* B6 with yellow stars, and *Ahl* *+* B6 and *Ahl* *+* B6.CBA with blue stars. Download Figure 3-1, TIF file.

10.1523/ENEURO.0465-24.2025.f3-2 Figure 3-2 **Network-level analysis of neuronal responses across genotypes.** **A,** Plot of the proportion of tone offset responses as a function of genotype, *n* = 8 mice for *ahl* and *Ahl* *+* B6, *n* = 5 for *Ahl* *+* B6.CBA. Two-way ANOVA (genotype: *F(2,15)* = 11.3, *p* = 0.001; sex: *F(1,15)* = 0.02, *p* = 0.89; interaction: *F(2,15)* = 1.18, *p* = 0.33), followed by post-hoc t-tests with Benjamini-Hochberg FDR correction. *: *p* < 0.05, **: *p* < 0.01 **B,** Plot of signal correlations among sound-responsive neurons as a function of genotype, *n* = 8 mice for *ahl* and *Ahl* *+* B6, *n* = 5 for *Ahl* *+* B6.CBA. Two-way ANOVA (genotype: *F(2,15)* = 16.7, *p* < 0.001; sex: *F(1,15)* = 0.42, *p* = 0.52; interaction: *F(2,15)* = 0.50, *p* = 0.61), followed by post-hoc t-tests with Benjamini-Hochberg FDR correction. **: *p* < 0.01, ***: *p* < 0.001. **C,** Plot of noise correlations among sound-responsive neurons as a function of genotype, *n* = 8 mice for *ahl* and *Ahl* *+* B6, *n* = 5 for *Ahl* *+* B6.CBA. Two-way ANOVA (genotype: *F(2,15)* = 0.06, *p* = 0.93; sex: *F(1,15)* = 1.11, *p* = 0.31; interaction: *F(2,15)* = 0.97, *p* = 0.40). **D,** Plot of signal correlations among sound-responsive neurons as a function of genotype, conditioned on low frequencies (4, 8, 16 kHz) and higher sound levels (70 and 90  dB SPL), *n* = 8 mice for *ahl* and *Ahl* *+* B6, *n* = 5 for *Ahl* *+* B6.CBA. Two-way ANOVA (genotype: *F(2,15)* = 41.0, *p* < 0.001; sex: *F(1,15)* = 3.28, *p* < 0.001; interaction: *F(2,15)* = 2.95, *p* = 0.08), followed by post-hoc t-tests with Benjamini-Hochberg FDR correction. **: *p* < 0.01, ***: *p* < 0.001. Download Figure 3-2, TIF file.

To assess how neurons in a network connect and organize, we calculated signal correlations, which measure the similarity in sound-evoked responses between pairs of sound-responsive neurons, and noise correlations, which reflect the similarity in activity that is independent of the stimuli. When averaging across all frequency and attenuation levels, we observed no significant differences between *Ahl+* B6 and *ahl* B6 mice in either signal or noise correlations (Extended Data Fig. 3-2*B*,*C*). However, *Ahl+* B6.CBA mice exhibited lower signal correlations than *Ahl+* or *ahl* B6 mice (Extended Data Fig. 3-2*B*), suggesting a sparser representation of tones in these mice (Bowen et al., 2020).

To understand how the response properties of individual neurons differ between genotypes, we calculated standard tuning properties for each neuron. The proportion of neurons with wide bandwidths, defined as the range in octaves between the minimum and maximum responsive frequency at 70 dB SPL (Fig. 3*E*), was higher in *Ahl+* B6 mice than in *ahl* B6 (Fig. 3*F*), consistent with the lack of response to 32 and 64 kHz tones in *ahl* B6 mice at this sound level (Fig. 3*D*). Similarly, *Ahl+* B6 mice exhibited higher proportions of neurons that responded with the highest amplitude to 64 kHz, regardless of the sound level (Fig. 3*G*, best frequency). Remarkably, when examining the characteristic frequency, defined as the frequency that elicited the highest response at the lowest sound level, neurons in *ahl* B6 mice exhibited a dramatic shift toward lower frequencies, with a significantly higher proportion of neurons with characteristic frequencies of 8 kHz and significantly lower proportions of neurons with characteristic frequencies of 32 and 64 kHz (Fig. 3*H*). Given similar numbers of sound-responsive neurons across genotypes (Fig. 3*C*), these data suggest that connections among neurons within *ahl* B6 mice had reorganized, allocating more cortical area to lower frequencies and reflecting the concurrent loss of high-frequency sensitivity in the inner ear.

### Reorganization of cortical responses toward lower frequencies in *ahl* B6 mice

**Figure 4.**
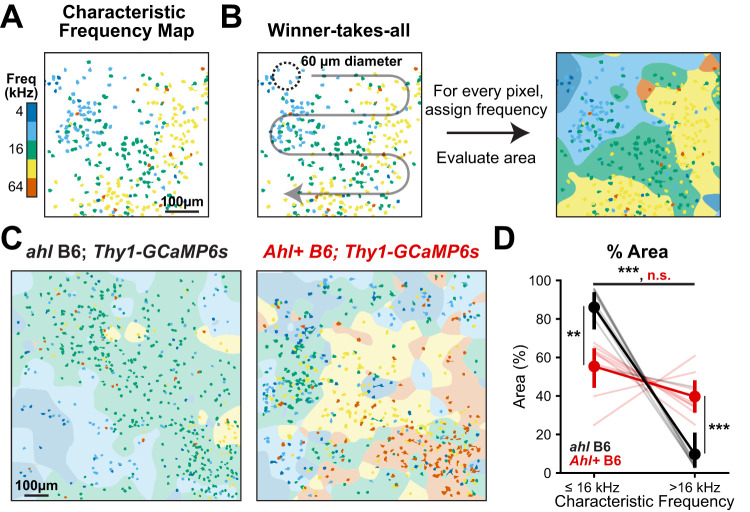
Reorganization of the auditory cortex toward lower frequencies in *ahl* B6 mice. ***A***, The plot of neuron location with characteristic frequencies indicated with color. ***B***, Schematic of the analytic method to quantify the cortical area devoted to processing a given characteristic frequency. A Gaussian filter (*σ* = 60 µm) was used to create a weighted sum of each neuron's characteristic frequency response for each unit of area. The frequency with the highest value was assigned to that unit of area and proportion of area examined. ***C***, Exemplar characteristic frequency maps and assigned areas for the indicated genotypes. ***D***, The plot of the fractional area as a function of characteristic frequency range and genotype. Light lines are individual animals; dark lines are mean ± SEM; *n* = 8 mice for each genotype. Two-way ANOVA (characteristic frequency, *F*_(1,36)_ = 72.98; *p* < 0.001; genotype, *F*_(2,36)_ = 0.08; *p* = 0.92; interaction, *F*_(2,36)_ = 34.6; *p* < 0.001), followed by post hoc *t* tests with Benjamini–Hochberg FDR correction. n.s., not significant; **p < 0.01; ***p < 0.001. Extended Data Figure 3-1 extends this analysis to include comparisons to *Ahl+* B6.CBA; *Thy1-GCaMP6s* mice.

To directly assess if reorganization of response properties occurred across the spatial extent of A1, we generated spatial characteristic frequency maps that mark the location and characteristic frequency of each neuron (Fig. 4*A*). Using a winner-takes-all approach, we summed the responses of local neurons exhibiting the same characteristic frequency using a Gaussian filter (*σ* = 60 µm) and assigned the frequency with the highest value to that unit of area (Fig. 4*B*). Compared with *Ahl+* B6 and *Ahl+* B6.CBA mice, which exhibited frequencies distributed across the field of view (Fig. 4*C*; Extended Data Fig. 3-1*C*,*D*), *ahl* B6 mice displayed a remarkable shift toward processing lower frequencies (≤16 kHz), with a majority (86 ± 14%) of the cortical area responding best to that range. We then reexamined signal correlation measurements by conditioning the correlations on lower frequencies and higher sound levels, where *ahl* B6 mice are most likely to respond, and found that signal correlations were much higher (*r* mean ± STD, 0.16 ± 0.04) in *ahl* B6 mice compared with *Ahl+* B6 (0.10 ± 0.02; *t* test with corrected *p* = 0.004) and B6.CBA (0.03 ± 0.01; *t* test with corrected *p* < 0.001) mice (Extended Data Fig. 3-2*D*), consistent with a reorganization toward lower frequencies. This increase in signal correlations likely reflects increasing amounts of shared input from thalamocortical pathways or strengthened synaptic strength of spared inputs. We did not observe cortical areas devoid of auditory responses, which might be predicted after a sudden loss of high-frequency hearing (Noreña et al., 2010), suggesting that auditory circuits had already undergone plasticity and/or increased cortical gain in response to degraded high-frequency input (Willott et al., 1993; Chambers et al., 2016; McGill et al., 2022).

### Linear predictive model of population responses performs worse at high frequencies and low sound levels in *ahl* B6 mice

**Figure 5.**
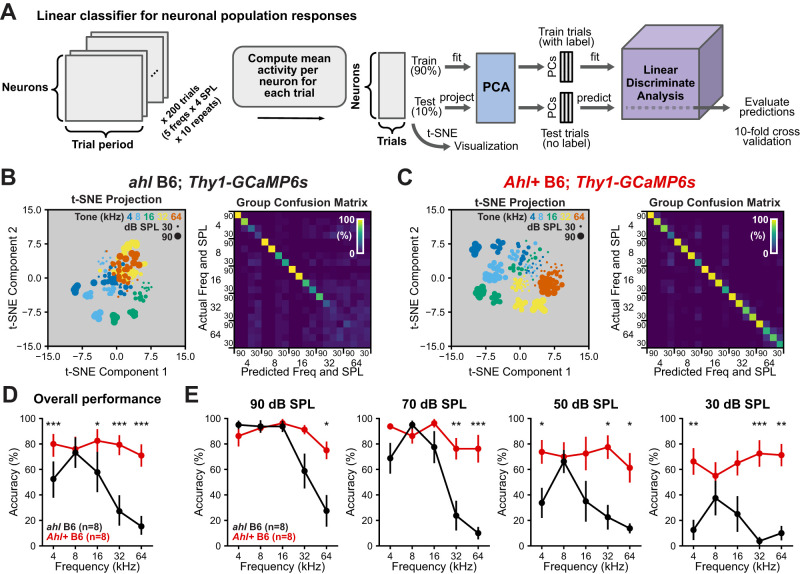
The linear classifier of network response performs worse in *ahl* B6 mice. ***A***, Schematic of a pipeline for prediction of frequency and the sound level based on population-level response using LDA. ***B***, ***C***, Left, Low-dimensional representation (*t*-SNE) of neuronal population response from an individual animal with each marker representing a single trial. Right, Confusion matrix of classifier performance from a single animal. ***D***, The plot of overall classifier performance as a function of frequency. Markers are mean ± SEM; *n* = 8 mice for each genotype. Three-way ANOVA (genotype, *F*_(2,360)_ = 76.7; *p* < 0.001; frequency, *F*_(4,360)_ = 15.2; *p* < 0.001; sound level, *F*_(3,360)_ = 47.8; *p* < 0.001; interaction, *F*_(50,360)_ = 2.89; *p* < 0.001), all attenuation levels included for each frequency in post hoc *t* tests with Benjamini–Hochberg FDR correction. *p < 0.01; **p < 0.01; ***p < 0.001. ***E***, The plot of classifier performance as a function of frequency (*x*-axis) and the sound level (plots arranged from highest sound level to lowest sound level). Markers are mean ± SEM; *n* = 8 mice for each genotype. The same three-way ANOVA as reported in ***D***, post hoc *t* tests with Benjamini–Hochberg FDR correction. *p < 0.01; **p < 0.01; ***p < 0.001. Extended Data Figure 3-1 extends this analysis to include comparisons to *Ahl+* B6.CBA; *Thy1-GCaMP6s* mice.

C57BL/6 mice are commonly used in behavioral experiments involving acoustic stimuli (Inagaki et al., 2018; Guo et al., 2019; Breton-Provencher et al., 2022), but our results suggest that these animals process sounds differently. While responses to high frequencies are much lower in *ahl* B6 mice, there remains a small population of neurons that do respond (Fig. 3*G*,*H*), suggesting that the cortex could decode those tones, such as in the case of mothers responding to pup calls (Marlin et al., 2015). To understand which frequencies and sound levels contain the most information, we designed a linear classifier to predict both the frequency and sound level being presented given linear features extracted from the population of neuronal responses (Fig. 5*A*). The population responses were first transformed into a lower-dimensional space using principal component analysis, before training a linear discriminate analysis model with 10-fold cross-validation. In *ahl* B6 mice, the population response contained less information relative to *Ahl+* B6 mice, as indicated by overlapping trial responses in lower-dimensional space and more incorrect predictions with 32 and 64 kHz tone classification (Fig. 5*B*). In contrast, *Ahl+* B6 and B6.CBA mice had well-separated clusters and a high percentage of correct predictions across frequency and sound levels (Fig. 5*C*; Extended Data Fig. 3-1*E*). Overall, the linear classifier performed significantly better at 4, 16, 32, and 64 kHz in *Ahl+* B6 mice when combining all attenuation levels (Fig. 5*D*). Examining accuracy as a function of the sound level, both models performed similarly at lower frequencies and above chance (5%) at higher frequencies at 90 dB SPL (Fig. 5*E*). At 50 and 70 dB SPL, accuracy at 32 and 64 kHz was much lower in *ahl* B6 but slightly above chance (Fig. 5*E*), indicating preservation of information at these sound levels. Information at 8 and 16 kHz was largely preserved across all sound levels across mice, with no statistical significance between the groups between 30 and 90 dB SPL (Fig. 5*E*). Similarly, *ahl* B6 mice exhibit highly predictive neural responses to 4 kHz at 70 and 90 dB SPL, with less predictive power at lower sound levels compared with *Ahl+* B6 mice, consistent with weak responses observed with widefield and two-photon imaging (Figs. 1*E*, 3*D*). Taken together, these data suggest that loud sounds can evoke discriminative properties in population neuronal responses in *ahl* B6 mice, while softer sounds cannot. Therefore, behavioral experiments using *ahl* B6 mice could be confounded by diminished peripheral and cortical responses to low sound levels.

### *Ahl+* B6 mice retain hearing sensitivity at 6 months of age, while *ahl* B6 mice lose it

**Figure 6.**
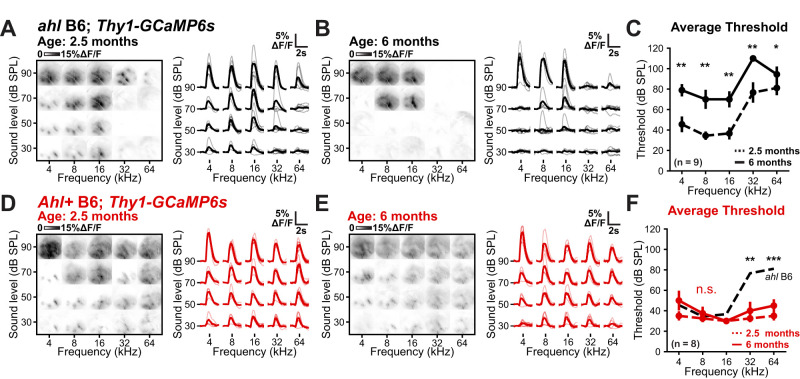
Low-threshold responses are intact in 6-month-old *Ahl+* B6 mice. ***A***, Left, The plot of fluorescence changes in 2.5-month-old *ahl* B6 mice over the imaging field to varying frequency (*x*-axis) and sound levels (*y*-axis). Right, The plot of fluorescence during sound presentation across animals; gray traces are individual mice; black traces are the average; *n* = 9 mice. ***B***, The plot of fluorescence changes in 6-month-old *ahl* B6 mice over the imaging field to varying frequency (*x*-axis) and sound levels (*y*-axis). Right, The plot of fluorescence during sound presentation across animals; gray traces are individual mice; black is the average; *n* = 9 mice. Note the complete disappearance of responses to 32 and 4–16 kHz at low sound levels. ***C***, The plot of average threshold as a function of frequency and time point. Dashed lines indicate measurements at 2.5 months; solid lines indicate measurements at 6 months; *n* = 9 mice. Markers at 110 dB SPL indicate no response was observed at 90 dB SPL. Three-way ANOVA (frequency, *F*_(4,70)_ = 10.6; *p* < 0.001; timepoint, *F*_(1,70)_ = 97.8; *p* < 0.001; sex, *F*_(1,70)_ = 0.29; *p* = 0.59; interaction, *F*_(13,70)_ = 1.51; *p* = 0.14) with post hoc paired *t* tests with Benjamini–Hochberg FDR correction. *p < 0.01; **p < 0.01; ***p < 0.001. ***D***, Similar to ***A***, but for *Ahl+* B6 mice; *n* = 8 mice. ***E***, Similar to ***B***, but for *Ahl+* B6 mice. *n* = 8 mice. ***F***, Similar to ***C***, but for *Ahl+* B6 mice. *n* = 8 mice. Three-way ANOVA (frequency, *F*_(4,60)_ = 2.26; *p* = 0.07; timepoint, *F*_(1,60)_ = 7.83; *p* = 0.007; sex, *F*_(1,60)_ = 0.29; *p* = 0.009; interaction, *F*_(13,60)_ = 0.71; *p* = 0.74) with post hoc paired *t* tests with Benjamini–Hochberg FDR correction. n.s., not significant.

Researchers must invest significant time to train mice on behavioral tasks. Given the progressive nature of hearing loss in C57BL/6 mice, mice could experience varying hearing sensitivity over the training period. To quantify the degree of this hearing loss, we performed widefield imaging on the same cohort of mice at 6 months of age (Fig. 6*A,B*). In *ahl* B6 mice, there was a near-total loss of sensitivity to tones presented at both 30 and 50 dB SPL (Fig. 6*B*). The thresholds for detecting a sound-evoked response significantly increased across all frequencies tested (ANOVA followed by *t* tests with corrected *p* values: *p* < 0.001, *p* < 0.001, *p* = 0.002, *p* = 0.007, *p* = 0.012 for 4, 8, 16, 32, and 64 kHz, respectively), with most mice exhibiting no sound-evoked response for 32 or 64 kHz tones at the highest sound level presented (90 dB; Fig. 6*B*,*C*). *Ahl+* B6 and B6.CBA mice retained sound-evoked responses across the entire frequency and attenuation range tested, with no significant differences observed in sound-evoked response thresholds (Fig. 6*D*–*F*; Extended Data Fig. 1-1*D–F*). Moreover, 6-month-old *Ahl+* B6 and B6.CBA thresholds were no different or lower than 2.5-month-old *ahl* B6 mice (Fig. 6*F*; Extended Data Fig. 1-1*F*). While *Ahl+* B6 mice exhibited 6 month thresholds that were not statistically significant from 2.5 month thresholds, there was a trend toward less sensitivity at the lowest and highest frequencies tested (Fig. 6*F*). *Ahl+* B6.CBA mice do not show this same loss of sensitivity (Extended Data Fig. 1-1*F*), consistent with ABR studies that indicate that *Ahl+* B6 mice have slightly elevated hearing thresholds compared with outbred mice (Kane et al., 2012). Together, these data indicate that *Ahl+* B6 mice retain and *ahl* B6 lose a majority of their hearing sensitivity in the first 6 months of life.

## Discussion

Transgenic mice are transformative tools that allow precise manipulation and visualization of neural circuits. Because many commonly used transgenic mice are maintained on a C57BL/6 genetic background (Dana et al., 2014; de Vries et al., 2020), they exhibit progressive hearing loss (Mikaelian et al., 1974), acting as a potential confound in studies examining many aspects of brain function. Common strategies used to circumvent this deficit, such as generating CRISPR/Cas9 single-nucleotide variants or congenic mice, are time- and resource-intensive and simply not feasible for most laboratories.

**Figure 7.**
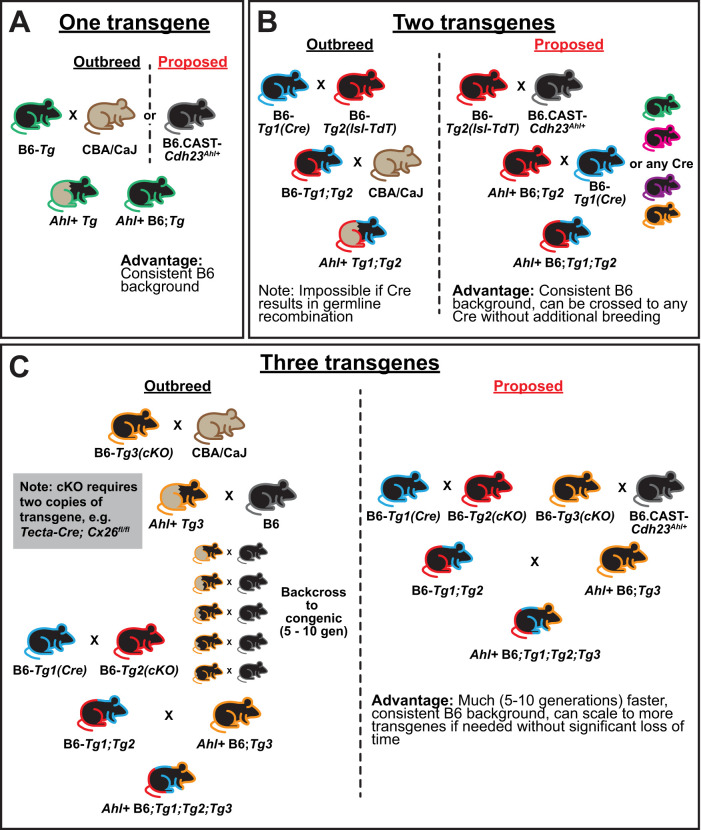
Breeding strategies to preserve hearing in transgenic mice. ***A***, Schematic of breeding strategy for a single transgene, e.g., *Thy1-GCaMP6s* used in this study. Mouse fill indicates its strain; black is C57BL/6; brown is CBA/CaJ; brown/black indicates mixed strain. Colored outlines indicate the transgene. ***B***, Schematic of breeding strategy for two transgenes, e.g., *Cre* and Cre-dependent (lox-stop-lox; lsl) reporter. Placing the reporter transgene on *Ahl+* background allows easy crossing to multiple Cre lines. ***C***, Schematic of breeding strategy for three transgenes, e.g., *Cre* and conditional knock-out (cKO) alleles (floxed/floxed).

Here, we highlight a simple and cost-effective strategy to generate transgenic C57BL/6 mice with limited hearing loss. By crossing commercially available congenic B6.CAST-*Cdh23*^Ahl+^/Kjn mice to pan-neuronal *Thy1-GCaMP6s* mice, we generated offspring with the *Ahl+* allelic variant, known to limit progressive hearing loss (Keithley et al., 2004). These mice exhibited low thresholds to high-frequency tones and retained these thresholds at 6 months of age, similar to mice outbred to the CBA/CaJ strain (Fig. 6; Extended Data Fig. 1-1). In contrast, mice without this variant (C57BL/6) exhibited elevated thresholds to high frequencies in early adulthood (Figs. 1, 3), reorganization of the auditory cortex to respond best to low-frequency tones (Fig. 4), and progressive loss of sensitivity in the first half-year of life (Fig. 6). We show that this variant is easy to genotype with traditional PCR and restriction enzyme digest, preventing the need for DNA purification and sequencing (Fig. 2). This strategy is scalable to more complex genetic strategies, such as conditional knock-outs or other models requiring multiple transgenes (Fig. 7), enabling more interpretable studies in neuroscience.

### C57BL/6 mice exhibit progressive hearing loss

Macroscopic widefield and two-photon imaging of *Thy1-GCaMP6s* mice expressing pan-neuronal GCaMP revealed an absence of low-threshold, cortical responses to high-frequency sounds when mice were generated on a C57BL/6 background (Figs. 1, 3). Previous studies have indicated that C57BL/6 mice contain an allelic variant in *Cdh23* that is responsible for the degradation of auditory responses and loss of hair cells, which is prevented by introducing a *Cdh23^c.753A^ ^>^ ^G^* single-nucleotide substitution (Johnson et al., 2017) or by outbreeding and selecting for the *Ahl+* locus (Kane et al., 2012). Consistent with these observations, we observed robust low-threshold responses at high frequencies (32 and 64 kHz) by crossing *Thy1-GCaMP6s* mice to either *Ahl+* B6 mice (B6.CAST-Cdh23^Ahl+^/Kjn) or *Ahl+* CBA/CaJ mice. While responses to high frequencies were diminished on a pure C57BL/6 background, they were not absent as indicated by responses to tones presented at elevated sound levels (Fig. 1*E*) and nonzero proportions of individual neurons exhibiting characteristic frequencies in that range (Fig. 3*H*). These data are consistent with single-unit recordings in the primary auditory cortex of breeding age (8–12 weeks old) C57BL/6 females, which respond to pup calls that contain frequency components exclusively in the ultrasonic range (Marlin et al., 2015).

Two-photon imaging of cortical neurons in 2.5-month-old C57BL/6 mice revealed significant reorganization of neuronal tuning, with most neurons selectively responding to tones ≤16 kHz (Fig. 4*C*,*D*). These data align with tonotopic map plasticity observed in cortical neurons, as measured with single-unit recordings in 3-month-old C57BL/6 mice (Willott et al., 1993). Similarly, ABRs recorded from 3-week-old C57BL/6 mice display elevated thresholds at high frequencies compared with CBA/CaJ controls (Zhang et al., 2013), with other studies indicating hearing deficits as early as 3 months of age (Zheng et al., 1999; Ouagazzal et al., 2006; Kane et al., 2012). These data strongly suggest that substantial hearing loss has already occurred by early adulthood, prompting plasticity of the remaining sensory input. Additionally, cortical responses to tones decline sharply over the next 4 months in C57BL/6 mice (Fig. 6*A–C*), which may prompt further reorganization due to reduced auditory drive (Willott et al., 1993). The progressive nature of this hearing loss inherently means that each mouse experiences a varying degree of hearing impairment during the first few months of life, a critical period for many experiments, raising concerns about its role as a potential confounding factor in many other studies.

### Potential confounds for systems neuroscience experiments

The use of C57BL/6 mice for auditory physiology and behavioral tasks has clear limitations. If the mice cannot hear or have an altered perception of the sound as part of the task, low performance may be mistakenly attributed to cognitive or motivational deficits, rather than a sensory deficit. For example, auditory cues are often paired with rewards in behavioral experiments (Olsen and Winder, 2009; Guo et al., 2019), and hearing loss can disrupt this association. If two tones are used, e.g., one low (16 kHz) and one high (32 kHz), behavioral differences at these two frequencies may reflect hearing loss, ultimately leading to incorrect assessments of reward-related learning without proper controls.

Progressive hearing loss can also impact behavioral experiments that use auditory cues to trigger a behavior (Inagaki et al., 2018; Li et al., 2021; Robert et al., 2021; Breton-Provencher et al., 2022). While the loss of peripheral input is a concern, this deficit could result in large-scale cross–modal plasticity (see Lee and Whitt, 2015 for review), with a decreased peripheral drive leading to compensatory changes across sensory systems (Petrus et al., 2014). Indeed, humans who experience deafness early in life display higher visual attention and processing, correlated with higher visually related activity within the anatomical auditory cortex (Dye and Bavelier, 2013). Likewise, hearing deficits could introduce compensatory behaviors, such as a reliance on or adaptation to other sensory cues that will ultimately disrupt the interpretation of those studies.

Pup-rearing behavior may also be impacted by hearing loss. During isolation or distress, newborn mouse pups signal their mothers with ultrasonic calls (>40 kHz). In C57BL/6 mice, first-time mothers exhibited more reliable and temporally precise action potential firing in response to these calls compared with virgin females (Marlin et al., 2015). While these findings might seem inconsistent with this study, pup distress calls can reach 80–90 dB SPL (Ehret, 2013), a sound level sufficient to elicit neural responses in *ahl* B6 mice (Fig. 1*E*). However, variability in the prior study may reflect different degrees of hearing loss in the maternal population. Additionally, C57BL/6 mothers take longer to retrieve pups than CBA/CaJ mothers (Stevenson et al., 2021) and do not retrieve vocalizing pups at higher rates than nonvocalizing pups (Winters et al., 2023), indicating that other cues, such as pup odor (Okabe et al., 2013; Vinograd et al., 2017) or lower-frequency calls (Ehret, 2006), may play a larger role in pup retrieval behaviors in C57BL/6 mice.

Environmental awareness is also largely driven by hearing, and loss of this sensation has been correlated with an increase in anxiety and stress levels in rodents (Lauer et al., 2018), potentially influencing behavioral responses in a range of experimental paradigms. Moreover, in humans, hearing deficits are linked to social isolation, depression, and dementia (Garnefski and Kraaij, 2012; Mick et al., 2014; Blazer and Tucci, 2019), and behavioral correlates may exist in mice. Together, these studies indicate that the potential for hearing loss as a confounding factor impacts systems neuroscience as a whole, not just the auditory field. Therefore, researchers must carefully consider their choice of animal model and the potential impact of sensory deficits on their experimental outcomes to avoid misleading interpretations.

### Practical considerations for experiments

When using congenic *Ahl+* B6 mice to generate transgenic mouse models, we offer several suggestions. With a single transgene, the transgenic mouse can simply be crossed to B6.CAST-*Cdh23*^Ahl+^/Kjn rather than outbreeding to CBA/CaJ to maintain a consistent genetic background (Fig. 7*A*). In most cases, we recommend breeding the mouse of interest to *Ahl+* homozygosity (*Cdh23*^Ahl/Ahl^). One allele is sufficient to protect against age-related hearing loss (Perrin et al., 2013; Mianné et al., 2016), so all offspring from these breeders will retain low-threshold hearing. Maintaining breeders with *Cdh23*^Ahl/Ahl^ also limits extensive genotyping, as the offspring genotypes are predetermined. In experiments requiring Cre and Cre-dependent reporters, placing *Ahl+* alleles on the reporter line is usually advantageous, because those reporters can be used with multiple Cre lines (Fig. 7*B*), as opposed to separately breeding those alleles onto each Cre line of interest. However, in cases of BAC transgenic Cre lines (which are generated with random insertion of Cre mediated by a bacterial artificial chromosome), it could be faster to reach homozygosity (three total alleles) than in reporter lines (four alleles, assuming homozygosity in *Cdh23* and reporter). When conditional deletion is required, our only suggestion is to reach *Ahl+* homozygosity on whichever mouse line has fewer total alleles to minimize the amount of total breeding required to generate the desired genotype (Fig. 7*C*).

While progressive hearing loss is a major factor in implementing this strategy, we also expect less variation in behavior, as previous studies have demonstrated remarkable differences among mouse strains in common behavioral tasks (Brooks et al., 2005; Kim et al., 2017) and brain plasticity (Ranson et al., 2013). In this study, we observed that *Ahl+* B6.CBA mice exhibited lower signal correlations and higher proportions of the tone offset responding neurons than either *ahl* or *Ahl+* B6 mice (Extended Data Fig. 3-2), indicating that these circuits operate differently than in C57BL/6 mice (Bowen et al., 2020). Ultimately, these differences would make comparisons between experiments using different strains extremely difficult.

We acknowledge that this approach requires startup costs related to obtaining, breeding, and ongoing maintenance of these mouse lines. However, the benefit of reduced variability in animal hearing will improve the interpretability of any study design by minimizing the risk of hearing loss as a confounding factor. In cases where this strategy is not feasible and C57BL/6 mice must be used, we strongly recommend using conventional hearing assessments (ABR) to include as an explanatory variable in any statistical models comparing groups, as in vivo contexts often rely on small sample sizes (∼5–6 mice/group) that can be significantly impacted by individual variability.

In summary, our work presents a streamlined strategy to mitigate progressive hearing loss in transgenic C57BL/6 mice. By introducing the *Ahl+* allelic variant through a cross with commercially available congenic B6.CAST-Cdh23^Ahl+^/Kjn mice, we observed limited progressive hearing loss, as evidenced by their sustained low-threshold responses to high-frequency sounds up to 6 months of age. These approaches are easy to implement, scalable to more complex genetic models, and eliminate the need for labor-intensive genotyping techniques, thereby improving the reliability and interpretability of research across multiple neuroscience disciplines.

## Synthesis

Reviewing Editor: Christine Portfors, Washington State University Decisions are customarily a result of the Reviewing Editor and the peer reviewers coming together and discussing their recommendations until a consensus is reached. When revisions are invited, a fact-based synthesis statement explaining their decision and outlining what is needed to prepare a revision will be listed below. The following reviewer(s) agreed to reveal their identity: Jinxing Wei. Note: If this manuscript was transferred from JNeurosci and a decision was made to accept the manuscript without peer review, a brief statement to this effect will instead be what is listed below.

The reviewers found the manuscript to be addressing an important topic as so many researchers use C57 mice even though they have age-related high frequency hearing loss. Some minor comments should be addressed, most importantly an explanation for why the thresholds at 32 and 64 kHz are so low as typically mouse audiograms are "U" shaped.

Specific comments:

The study missed an important opportunity to examine auditory brainstem responses (ABRs) in the same mice from which cortical signals were recorded from. This is particularly relevant because of the rather low thresholds seen for 32 kHz and 64 kHz responses in Ahl+ mice. For example Figure panels 1I, 3D (maybe even Fig 6F at 6 mo) shows a flat threshold curve for frequencies tested, including the really high frequency tones. This is surprising even if the data shown are averages across sites/mice. But mouse audiograms are typically 'U' shaped, with higher thresholds for 32 kHz, 64 kHz tones. So, this mismatch is a bit confusing.

Minor comment:

Some important older citations can be added.

Vázquez, A.E., Jimenez, A.M., Martin, G.K., Luebke, A.E. and Lonsbury-Martin, B.L., 2004. Evaluating cochlear function and the effects of noise exposure in the B6. CAST+ Ahl mouse with distortion product otoacoustic emissions. Hearing research, 194(1-2), pp.87-96.

Mock, B.E., Vijayakumar, S., Pierce, J., Jones, T.A. and Jones, S.M., 2016. Differential effects of Cdh23753A on auditory and vestibular functional aging in C57BL/6J mice. Neurobiology of aging, 43, pp.13-22.

Johnson, K.R., Erway, L.C., Cook, S.A., Willott, J.F. and Zheng, Q.Y., 1997. A major gene affecting age-related hearing loss in C57BL/6J mice. Hearing research, 114(1-2), pp.83-92.

In the Results section (line 323), the description of AAF is not entirely accurate. The authors need to provide clarification.

In Fig. 3D, the labeled neurons in Ahl+ B6 mice increased by 20% compared to Ahl B6 mice. Could this be due to higher labeling efficiency in the former? Is this difference statistically significant?

In Fig. S1F, there is a labeling error that needs to be corrected.

## References

[B1] Blazer DG, Tucci DL (2019) Hearing loss and psychiatric disorders: a review. Psychol Med 49:891–897. 10.1017/S0033291718003409 30457063

[B2] Bowen Z, Winkowski DE, Kanold PO (2020) Functional organization of mouse primary auditory cortex in adult C57BL/6 and F1 (CBAxC57) mice. Sci Rep 10:10905. 10.1038/s41598-020-67819-4 32616766 PMC7331716

[B3] Breton-Provencher V, Drummond GT, Feng J, Li Y, Sur M (2022) Spatiotemporal dynamics of noradrenaline during learned behaviour. Nature 606:732–738. 10.1038/s41586-022-04782-2 35650441 PMC9837982

[B4] Brooks SP, Pask T, Jones L, Dunnett SB (2005) Behavioural profiles of inbred mouse strains used as transgenic backgrounds. II: cognitive tests. Genes Brain Behav 4:307–317. 10.1111/j.1601-183X.2004.00109.x 16011577

[B5] Burghard AL, Morel NP, Oliver DL (2019) Mice heterozygous for the *Cdh23/Ahl1* mutation show age-related deficits in auditory temporal processing. Neurobiol Aging 81:47–57. 10.1016/j.neurobiolaging.2019.02.029 31247458 PMC6732241

[B6] Chambers AR, Resnik J, Yuan Y, Whitton JP, Edge AS, Liberman MC, Polley DB (2016) Central gain restores auditory processing following near-complete cochlear denervation. Neuron 89:867–879. 10.1016/j.neuron.2015.12.041 26833137 PMC4760846

[B7] Dana H, Chen T-W, Hu A, Shields BC, Guo C, Looger LL, Kim DS, Svoboda K (2014) Thy1-GCaMP6 transgenic mice for neuronal population imaging in vivo. PLoS One 9:e108697. 10.1371/journal.pone.0108697 25250714 PMC4177405

[B8] de Vries SEJ, et al. (2020) A large-scale standardized physiological survey reveals functional organization of the mouse visual cortex. Nat Neurosci 23:138–151. 10.1038/s41593-019-0550-9 31844315 PMC6948932

[B9] Dye MWG, Bavelier D (2013) Visual attention in deaf humans: a neuroplasticity perspective. In: *Deafness* (Kral A, Popper AN, Fay RR, eds), pp 237–263. New York, NY: Springer.

[B58] Ehret G (2006) Common rules of communication sound perception. In: *Behaviour and neurodynamics for auditory communication* (Kanwal JS, Ehret G, eds), pp 85–114. Cambridge: Cambridge University Press.

[B10] Ehret G (2013) Sound communication in house mice: emotions in their voices and ears? In: *Evolution of emotional communication: from sounds in nonhuman mammals to speech and music in man* (Altenmüller E, Schmidt S, Zimmermann E, eds), pp 63–74. Oxford, UK: Oxford University Press.

[B11] Frisina RD, Singh A, Bak M, Bozorg S, Seth R, Zhu X (2011) F1 (CBA × C57) mice show superior hearing in old age relative to their parental strains: hybrid vigor or a new animal model for “Golden Ears”? Neurobiol Aging 32:1716–1724. 10.1016/j.neurobiolaging.2009.09.009 19879021 PMC2891213

[B12] Garnefski N, Kraaij V (2012) Cognitive coping and goal adjustment are associated with symptoms of depression and anxiety in people with acquired hearing loss. Int J Audiol 51:545–550. 10.3109/14992027.2012.675628 22537001

[B13] Guo L, Weems JT, Walker WI, Levichev A, Jaramillo S (2019) Choice-selective neurons in the auditory cortex and in its striatal target encode reward expectation. J Neurosci 39:3687–3697. 10.1523/JNEUROSCI.2585-18.2019 30837264 PMC6510333

[B14] International Mouse Knockout Consortium (2007) A mouse for all reasons. Cell 128:9–13. 10.1016/j.cell.2006.12.018 17218247

[B15] Inagaki HK, Inagaki M, Romani S, Svoboda K (2018) Low-dimensional and monotonic preparatory activity in mouse anterior lateral motor cortex. J Neurosci 38:4163–4185. 10.1523/JNEUROSCI.3152-17.2018 29593054 PMC6596025

[B16] Ison J, Allen P (2004) Low-frequency tone pips elicit exaggerated startle reflexes in C57BL/6J mice with hearing loss. J Assoc Res Otolaryngol 4:495–504. 10.1007/s10162-002-3046-2 12784135 PMC3202743

[B17] Johnson KR, Erway LC, Cook SA, Willott JF, Zheng QY (1997) A major gene affecting age-related hearing loss in C57BL/6J mice. Hear Res 114:83–92. 10.1016/S0378-5955(97)00155-X 9447922

[B18] Johnson KR, Tian C, Gagnon LH, Jiang H, Ding D, Salvi R (2017) Effects of Cdh23 single nucleotide substitutions on age-related hearing loss in C57BL/6 and 129S1/Sv mice and comparisons with congenic strains. Sci Rep 7:44450. 10.1038/srep44450 28287619 PMC5347380

[B19] Kane KL, Longo-Guess CM, Gagnon LH, Ding D, Salvi RJ, Johnson KR (2012) Genetic background effects on age-related hearing loss associated with Cdh23 variants in mice. Hear Res 283:80–88. 10.1016/j.heares.2011.11.007 22138310 PMC3277672

[B21] Keithley EM, Canto C, Zheng QY, Fischel-Ghodsian N, Johnson KR (2004) Age-related hearing loss and the ahl locus in mice. Hear Res 188:21–28. 10.1016/S0378-5955(03)00365-4 14759567 PMC2858220

[B22] Kim JW, Nam SM, Yoo DY, Jung HY, Kim IY, Hwang IK, Seong JK, Yoon YS (2017) Comparison of adult hippocampal neurogenesis and susceptibility to treadmill exercise in nine mouse strains. Neural Plast 2017:5863258. 10.1155/2017/5863258 29391953 PMC5748094

[B23] Lauer AM, Larkin G, Jones A, May BJ (2018) Behavioral animal model of the emotional response to tinnitus and hearing loss. J Assoc Res Otolaryngol 19:67–81. 10.1007/s10162-017-0642-8 29047013 PMC5783924

[B24] Lee H-K, Whitt JL (2015) Cross-modal synaptic plasticity in adult primary sensory cortices. Curr Opin Neurobiol 35:119–126. 10.1016/j.conb.2015.08.002 26310109 PMC4641772

[B25] Li Z, Wei J-X, Zhang G-W, Huang JJ, Zingg B, Wang X, Tao HW, Zhang LI (2021) Corticostriatal control of defense behavior in mice induced by auditory looming cues. Nat Commun 12:1040. 10.1038/s41467-021-21248-7 33589613 PMC7884702

[B26] Liu J, Kanold PO (2021) Diversity of receptive fields and sideband inhibition with complex thalamocortical and intracortical origin in L2/3 of mouse primary auditory cortex. J Neurosci 41:3142–3162. 10.1523/JNEUROSCI.1732-20.2021 33593857 PMC8026349

[B27] Liu J, Whiteway MR, Sheikhattar A, Butts DA, Babadi B, Kanold PO (2019) Parallel processing of sound dynamics across mouse auditory cortex via spatially patterned thalamic inputs and distinct areal intracortical circuits. Cell Rep 27:872–885.e7. 10.1016/j.celrep.2019.03.069 30995483 PMC7238664

[B28] Luo L, et al. (2020) Optimizing nervous system-specific gene targeting with Cre driver lines: prevalence of germline recombination and influencing factors. Neuron 106:37–65.e5. 10.1016/j.neuron.2020.01.008 32027825 PMC7377387

[B29] Madisen L, et al. (2010) A robust and high-throughput Cre reporting and characterization system for the whole mouse brain. Nat Neurosci 13:133–140. 10.1038/nn.2467 20023653 PMC2840225

[B30] Marlin BJ, Mitre M, D’amour JA, Chao MV, Froemke RC (2015) Oxytocin enables maternal behaviour by balancing cortical inhibition. Nature 520:499–504. 10.1038/nature14402 25874674 PMC4409554

[B31] McGill M, Hight AE, Watanabe YL, Parthasarathy A, Cai D, Clayton K, Hancock KE, Takesian A, Kujawa SG, Polley DB (2022) Neural signatures of auditory hypersensitivity following acoustic trauma. Elife 11:e80015. 10.7554/eLife.80015 36111669 PMC9555866

[B32] Mianné J, et al. (2016) Correction of the auditory phenotype in C57BL/6N mice via CRISPR/Cas9-mediated homology directed repair. Genome Med 8:16. 10.1186/s13073-016-0273-4 26876963 PMC4753642

[B33] Mick P, Kawachi I, Lin FR (2014) The association between hearing loss and social isolation in older adults. Otolaryngol Head Neck Surg 150:378–384. 10.1177/0194599813518021 24384545

[B34] Mikaelian DO, Warfield D, Norris O (1974) Genetic progressive hearing loss in the C57/M6 mouse: relation of behaviorial responses to cochlear anatomy. Acta Otolaryngol 77:327–334. 10.3109/00016487409124632 4835632

[B35] Mock BE, Vijayakumar S, Pierce J, Jones TA, Jones SM (2016) Differential effects of Cdh23753A on auditory and vestibular functional aging in C57BL/6J mice. Neurobiol Aging 43:13–22. 10.1016/j.neurobiolaging.2016.03.013 27255811 PMC4893173

[B36] Noben-Trauth K, Zheng QY, Johnson KR (2003) Association of cadherin 23 with polygenic inheritance and genetic modification of sensorineural hearing loss. Nat Genet 35:21–23. 10.1038/ng1226 12910270 PMC2864026

[B37] Noreña AJ, Moffat G, Blanc JL, Pezard L, Cazals Y (2010) Neural changes in the auditory cortex of awake Guinea pigs after two tinnitus inducers: salicylate and acoustic trauma. Neuroscience 166:1194–1209. 10.1016/j.neuroscience.2009.12.063 20096752

[B38] Okabe S, Nagasawa M, Kihara T, Kato M, Harada T, Koshida N, Mogi K, Kikusui T (2013) Pup odor and ultrasonic vocalizations synergistically stimulate maternal attention in mice. Behav Neurosci 127:432–438. 10.1037/a0032395 23544596

[B39] Olsen CM, Winder DG (2009) Operant sensation seeking engages similar neural substrates to operant drug seeking in C57 mice. Neuropsychopharmacology 34:1685–1694. 10.1038/npp.2008.226 19145223 PMC2720253

[B40] Ouagazzal A-M, Reiss D, Romand R (2006) Effects of age-related hearing loss on startle reflex and prepulse inhibition in mice on pure and mixed C57BL and 129 genetic background. Behav Brain Res 172:307–315. 10.1016/j.bbr.2006.05.018 16814879

[B41] Pachitariu M, Stringer C, Dipoppa M, Schröder S, Rossi LF, Dalgleish H, Carandini M, Harris KD (2017) Suite2p: beyond 10,000 neurons with standard two-photon microscopy. bioRxiv.

[B42] Perrin BJ, Strandjord DM, Narayanan P, Henderson DM, Johnson KR, Ervasti JM (2013) β-Actin and fascin-2 cooperate to maintain stereocilia length. J Neurosci 33:8114–8121. 10.1523/JNEUROSCI.0238-13.2013 23658152 PMC3718021

[B43] Petrus E, Isaiah A, Jones AP, Li D, Wang H, Lee H-K, Kanold PO (2014) Crossmodal induction of thalamocortical potentiation leads to enhanced information processing in the auditory cortex. Neuron 81:664–673. 10.1016/j.neuron.2013.11.023 24507197 PMC4023256

[B44] Ranson A, Sengpiel F, Fox K (2013) The role of GluA1 in ocular dominance plasticity in the mouse visual cortex. J Neurosci 33:15220–15225. 10.1523/JNEUROSCI.2078-13.2013 24048851 PMC6618404

[B45] Robert B, Kimchi EY, Watanabe Y, Chakoma T, Jing M, Li Y, Polley DB (2021) A functional topography within the cholinergic basal forebrain for encoding sensory cues and behavioral reinforcement outcomes. Elife 10:e69514. 10.7554/eLife.69514 34821218 PMC8654357

[B46] Romero S, Hight AE, Clayton KK, Resnik J, Williamson RS, Hancock KE, Polley DB (2020) Cellular and widefield imaging of sound frequency organization in primary and higher order fields of the mouse auditory cortex. Cereb Cortex 30:1603–1622. 10.1093/cercor/bhz190 31667491 PMC7132909

[B47] Sinclair JL, Barnes-Davies M, Kopp-Scheinpflug C, Forsythe ID (2017) Strain-specific differences in the development of neuronal excitability in the mouse. Hear Res 354:28–37. 10.1016/j.heares.2017.08.004 28843833

[B48] Stevenson P, Casenhiser DM, Lau BYB, Krishnan K (2021) Systematic analysis of goal-related movement sequences during maternal behavior in a female mouse model for Rett syndrome. Eur J Neurosci 54:4528–4549. 10.1111/ejn.15327 34043854 PMC8450021

[B49] Sultana R, Ogundele OM, Lee CC (2019) Contrasting characteristic behaviours among common laboratory mouse strains. R Soc Open Sci 6:190574. 10.1098/rsos.190574 31312505 PMC6599779

[B50] Vázquez AE, Jimenez AM, Martin GK, Luebke AE, Lonsbury-Martin BL (2004) Evaluating cochlear function and the effects of noise exposure in the B6.CAST+*Ahl* mouse with distortion product otoacoustic emissions. Hear Res 194:87–96. 10.1016/j.heares.2004.03.017 15276680

[B51] Vinograd A, Fuchs-Shlomai Y, Stern M, Mukherjee D, Gao Y, Citri A, Davison I, Mizrahi A (2017) Functional plasticity of odor representations during motherhood. Cell Rep 21:351–365. 10.1016/j.celrep.2017.09.038 29020623 PMC5643523

[B52] Willott JF, Aitkin LM, McFadden SL (1993) Plasticity of auditory cortex associated with sensorineural hearing loss in adult C57BL/6J mice. J Comp Neurol 329:402–411. 10.1002/cne.903290310 8459051

[B53] Winkowski DE, Kanold PO (2013) Laminar transformation of frequency organization in auditory cortex. J Neurosci 33:1498–1508. 10.1523/JNEUROSCI.3101-12.2013 23345224 PMC3783029

[B54] Winters C, Gorssen W, Wöhr M, D’Hooge R (2023) BAMBI: a new method for automated assessment of bidirectional early-life interaction between maternal behavior and pup vocalization in mouse dam-pup dyads. Front Behav Neurosci 17:1139254. 10.3389/fnbeh.2023.1139254 36935889 PMC10020184

[B55] Wong GT (2002) Speed congenics: applications for transgenic and knock-out mouse strains. Neuropeptides 36:230–236. 10.1054/npep.2002.0905 12359513

[B56] Zhang Q, Liu H, McGee J, Walsh EJ, Soukup GA, He DZZ (2013) Identifying microRNAs involved in degeneration of the organ of corti during age-related hearing loss. PLoS One 8:e62786. 10.1371/journal.pone.0062786 23646144 PMC3640032

[B57] Zheng QY, Johnson KR, Erway LC (1999) Assessment of hearing in 80 inbred strains of mice by ABR threshold analyses. Hear Res 130:94–107. 10.1016/S0378-5955(99)00003-9 10320101 PMC2855304

