# Supplementary figures and images for: Limiting Hearing Loss in Transgenic Mouse Models
Source: eNeuro. 2025 Feb 18;12(2):ENEURO.0465-24.2025. doi: 10.1523/ENEURO.0465-24.2025 (PMC11875052; doi:10.1523/ENEURO.0465-24.2025)

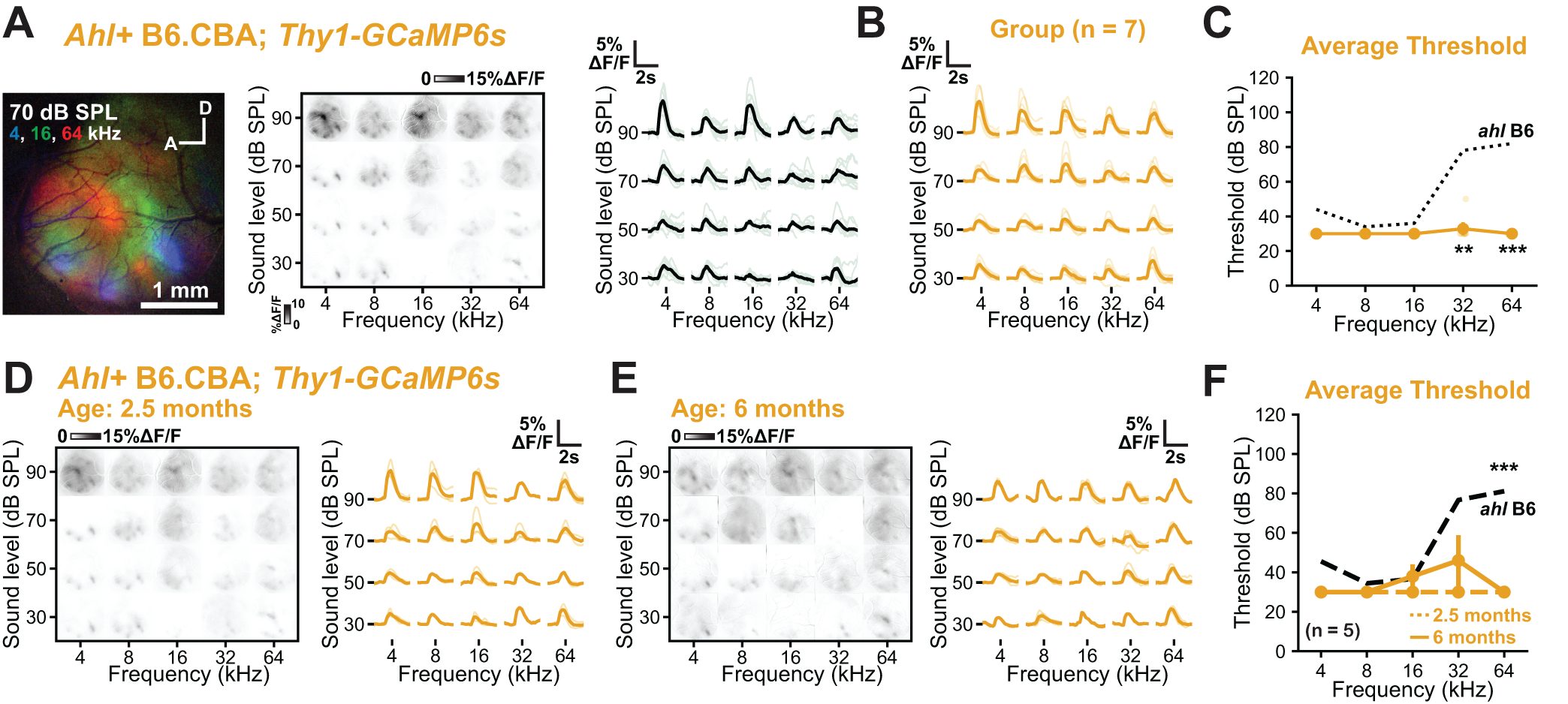

Supplement: Figure 1-1 — Widefield imaging in Ahl + B6.CBA mice reveals low threshold responses to a broad range of frequencies. A, (left) Merged fluorescence responses to 4 (blue), 16 (green), and 64 kHz (red) at 70 dB SPL in P70 Ahl + B6.CBA; Thy1-GCaMP6 s mice. (middle) Plot of fluorescence changes over the imaging field to varying frequency (x-axis) and sound levels (y-axis). (right) Plot of fluorescence during sound presentation from an individual mouse. Grey traces are individual trials, black traces are the average. B, Plot of fluorescence during sound presentation from all Ahl + B6.CBA; Thy1-GCaMP6 s mice. Light traces are individual mice, dark traces are the group average, n = 7 mice. C, Plot of average fluorescence detection threshold for each frequency presented, n = 7 mice. Three-way ANOVA (frequency: F(4,110) = 16.2, p = 1.8e-10; sex: F(1,110) = 4.76, p = 3.1e-02; genotype: F(2,110) = 61.7, p = 1.1e-18; interaction: F(22,110) = 5.13, p = 3.2e-09) followed by planned comparisons with t-tests controlled with Benjamini-Hochberg FDR. **: p < 0.01, ***: p < 0.001. D, (left) Plot of fluorescence changes in 2.5-month-old Thy1-GCaMP6 s (Ahl + B6.CBA) mice over the imaging field to varying frequency (x-axis) and sound levels (y-axis). (right) Plot of fluorescence during sound presentation across animals; grey traces are individual mice, black traces are the average, n = 5 mice. E, (left) Plot of fluorescence changes in 6-month-old Thy1-GCaMP6 s (Ahl + B6.CBA) mice over the imaging field to varying frequency (x-axis) and sound levels (y-axis). (right) Plot of fluorescence during sound presentation across animals; grey traces are individual mice, black traces are the average, n = 5 mice. F, Plot of average threshold as a function of frequency and time point. Dashed lines indicate measurements at 2.5 months, solid lines indicate measurements at 6 months, n = 5 mice. Three-way ANOVA (frequency: F(4,30) = 4.80, p = 4.1e-3; timepoint: F(1,30) = 10.8, p = 2.6e-3; sex: F(1,30) = 16.2, p = 3.6e- [file eneuro-12-ENEURO.0465-24.2025-s001.tif]

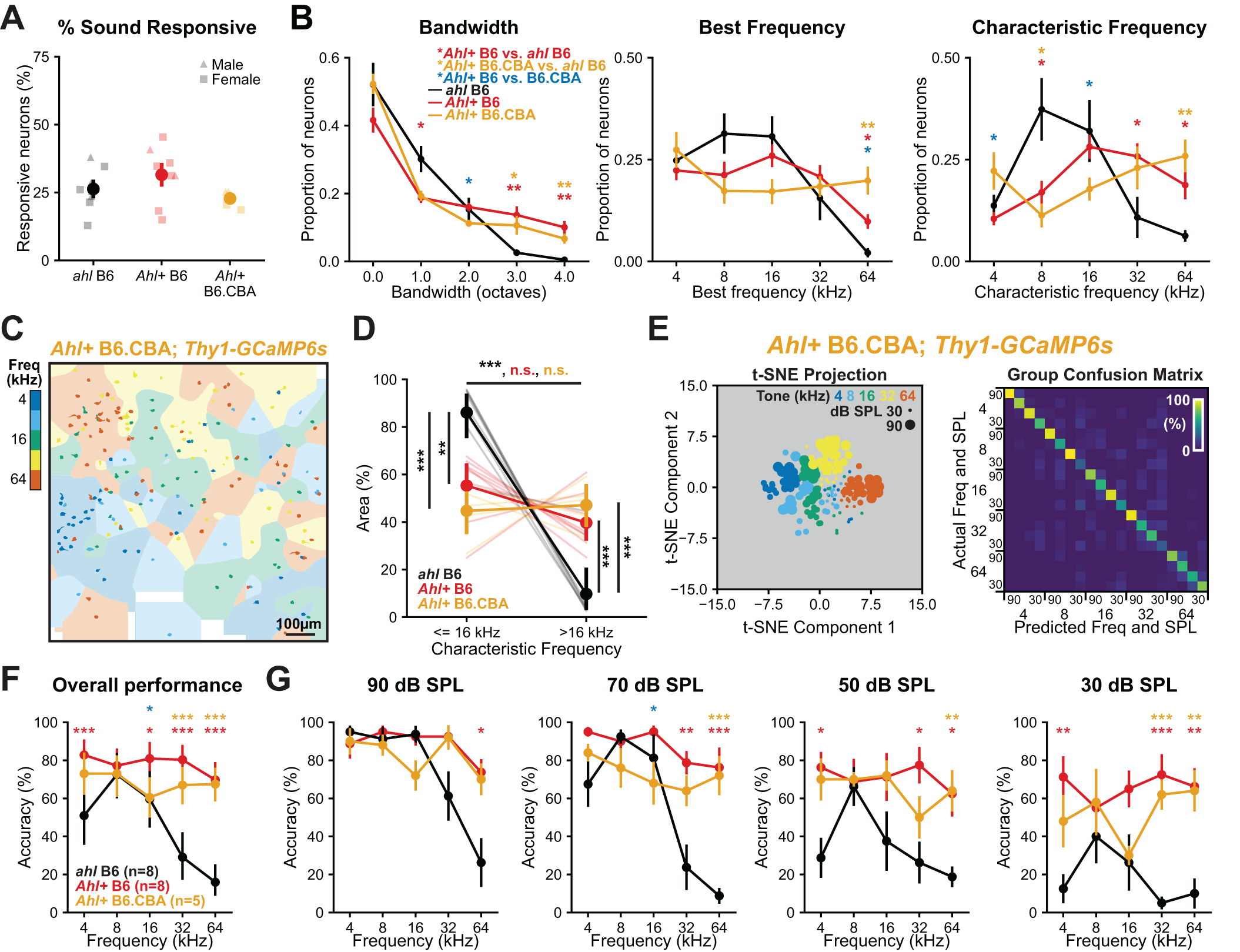

Supplement: Figure 3-1 — Neuronal responses in Ahl + B6.CBA are similar to Ahl + B6 mice. A, Plot of the proportion of sound-responsive neurons within each genotype. Light markers indicate individuals, dark markers are mean ± SEM, n = 8 mice for ahl and Ahl + B6 mice, n = 5 mice for Ahl + B6.CBA mice. Two-way ANOVA (genotype: F(2,15) = 2.42, p = 0.12; sex: F(1,15) = 1.95, p = 0.18, interaction: F(2,15) = 0.00, p = 0.99). n.s.: not significant. B, Plots of the proportion of neurons as a function of bandwidth, best frequency, and characteristic frequencies across genotypes. Points are mean ± SEM, n = 8 mice for ahl and Ahl + B6 mice, n = 5 mice for Ahl + B6.CBA mice. Two-way ANOVAs with genotype and measured characteristic, followed by post-hoc t-tests with Benjamini-Hochberg FDR correction. *: p < 0.05, **: p < 0.01, ***: p < 0.001. Comparisons between Ahl + B6 and ahl B6 are indicated with red stars, Ahl + B6.CBA and ahl B6 with yellow stars, and Ahl + B6 and Ahl + B6.CBA with blue stars. C, Exemplar characteristic frequency maps and assigned areas for Ahl + B6.CBA mice. White areas on the map indicate regions where no characteristic frequency was assigned due to a lack of neurons within the area. D, Plot of normalized area as a function of characteristic frequency and genotype. Light lines are individual animals, dark lines are mean ± SEM, n = 8 mice for ahl and Ahl + B6, n = 5 for Ahl + B6.CBA. Two-way ANOVA (characteristic frequency: F(1,36) = 72.98, p < 0.001; genotype: F(2,36) = 0.08, p = 0.92; interaction: F(2,36) = 34.6, p < 0.001), followed by post-hoc t-tests with Benjamini-Hochberg FDR correction. n.s: not significant, **: p < 0.01, ***: p < 0.001. E, (left) Low-dimensional representation (t-SNE) of neuronal population response from an individual Ahl + B6.CBA mouse with each marker representing a single trial. (right) Confusion matrix of classifier performance from a single animal. F, Plot of overall classifier performance as a function of frequency, n = 8 mice for ahl and Ahl + B [file eneuro-12-ENEURO.0465-24.2025-s002.tif]

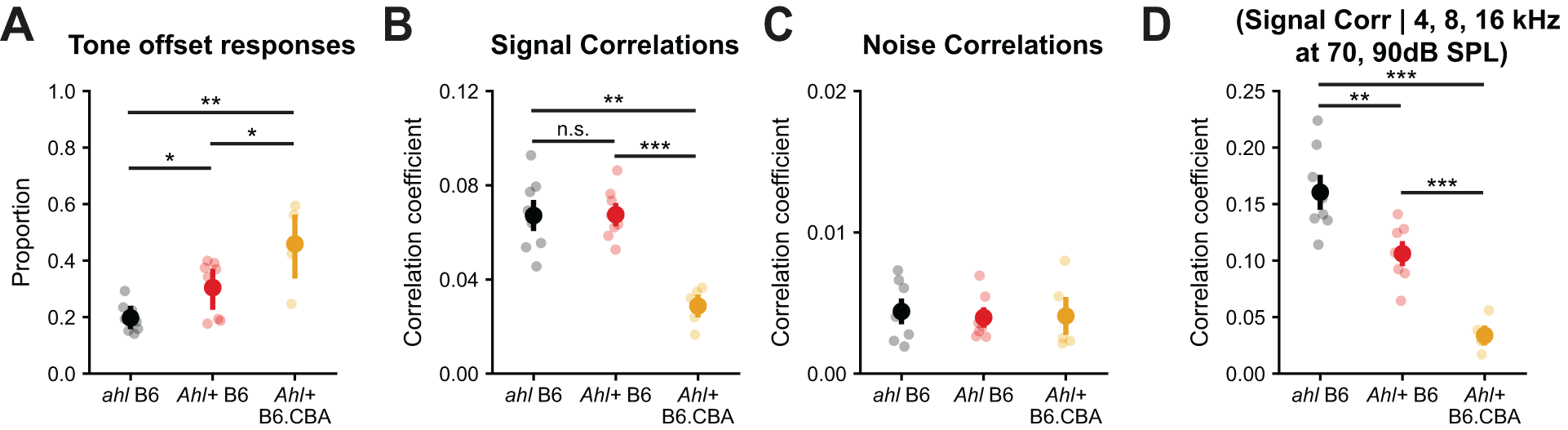

Supplement: Figure 3-2 — Network-level analysis of neuronal responses across genotypes. A, Plot of the proportion of tone offset responses as a function of genotype, n = 8 mice for ahl and Ahl + B6, n = 5 for Ahl + B6.CBA. Two-way ANOVA (genotype: F(2,15) = 11.3, p = 0.001; sex: F(1,15) = 0.02, p = 0.89; interaction: F(2,15) = 1.18, p = 0.33), followed by post-hoc t-tests with Benjamini-Hochberg FDR correction. *: p < 0.05, **: p < 0.01 B, Plot of signal correlations among sound-responsive neurons as a function of genotype, n = 8 mice for ahl and Ahl + B6, n = 5 for Ahl + B6.CBA. Two-way ANOVA (genotype: F(2,15) = 16.7, p < 0.001; sex: F(1,15) = 0.42, p = 0.52; interaction: F(2,15) = 0.50, p = 0.61), followed by post-hoc t-tests with Benjamini-Hochberg FDR correction. **: p < 0.01, ***: p < 0.001. C, Plot of noise correlations among sound-responsive neurons as a function of genotype, n = 8 mice for ahl and Ahl + B6, n = 5 for Ahl + B6.CBA. Two-way ANOVA (genotype: F(2,15) = 0.06, p = 0.93; sex: F(1,15) = 1.11, p = 0.31; interaction: F(2,15) = 0.97, p = 0.40). D, Plot of signal correlations among sound-responsive neurons as a function of genotype, conditioned on low frequencies (4, 8, 16 kHz) and higher sound levels (70 and 90 dB SPL), n = 8 mice for ahl and Ahl + B6, n = 5 for Ahl + B6.CBA. Two-way ANOVA (genotype: F(2,15) = 41.0, p < 0.001; sex: F(1,15) = 3.28, p < 0.001; interaction: F(2,15) = 2.95, p = 0.08), followed by post-hoc t-tests with Benjamini-Hochberg FDR correction. **: p < 0.01, ***: p < 0.001. Download Figure 3-2, TIF file. [file eneuro-12-ENEURO.0465-24.2025-s003.tif]
